# A Methodology for Deciphering the Transmembrane Resistance Variability of Supported Lipid Bilayers

**DOI:** 10.1002/advs.202508589

**Published:** 2025-11-10

**Authors:** Aristea Pavlou, Debdatta Panigrahi, Somayeh Kashani, Anna‐Maria Pappa, Fabrizio Torricelli, Paul W.M. Blom, Paschalis Gkoupidenis

**Affiliations:** ^1^ Max Planck Institute for Polymer Research Ackermannweg 10 55128 Mainz Germany; ^2^ Department of Electrical and Computer Engineering North Carolina State University 890 Oval Dr Raleigh NC 27606 USA; ^3^ Carbon Electronics Laboratories (ORaCEL) North Carolina State University Raleigh NC 27695 USA; ^4^ Department of Biomedical Engineering and Biotechnology Khalifa University Abu Dhabi PO Box 127788 UAE; ^5^ Department of Information Engineering University of Brescia via Branze 38 Brescia 25123 Italy; ^6^ Department of Physics North Carolina State University 2401 Stinson Dr Raleigh NC 27607 USA

**Keywords:** bioelectronics, biolipids, biomembrane variabilities, organic mixed‐conductors, supported lipid bilayers

## Abstract

In recent years, organic biomimetic electronic devices have gained attention for their potential applications in healthcare, as they emulate the natural functions of biological components that mediate communication between external signals and internal cellular processes. These devices integrate semi‐biological components such as synthetic membranes with organic electronics. For instance, Supported Lipid Bilayers (SLBs) offer a promising substrate as biomimetic membranes, by providing a stable and controlled interface for bioelectronic and biomimetic applications. However, challenges remain in SLB formation, particularly in achieving consistent transmembrane ionic resistance due to packing defects. This work reports a framework of the dielectric properties of a SLB dielectric stack, and investigates the impact of defects on the membrane resistance variations. According to the model, lipid packing non‐idealities lead to the partial hydration of the inner part of the membrane and thus to transmembrane resistance variations. These findings offer new insights into the dielectric and transmembrane barrier characteristics of SLBs by introducing a quantitative assessment method that transcends qualitative experimental observations, paving the way for a systematic approach to designing controllable membranes and biointerfaces with customizable biomimetic properties.

## Graphical abstract

This work shows that transmembrane resistance variability, due to lipid packing defects, can be quantified by analysing the dielectric properties of Supported Lipid Bilayers. The proposed methodology provides an analytical tool for optimizing SLB design with improved stability and functionality for bioelectronic and biomimetic applications.

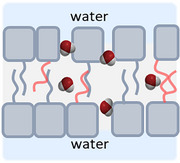

## 1 Introduction

Over the last decades, the fields of organic electronics and biology are increasingly intertwined, giving rise to the field of organic bioelectronics.^[^ ^1^, ^2^, ^3^ ^]^ Many advancements have been made in this continuously evolving field owing to its diverse applications, such as biosensors,^[^ ^4^ ^]^ drug delivery devices,^[^ ^5^ ^]^ organ‐on‐a‐chip devices,^[^ ^6^ ^]^ brain‐computer interfaces,^[^ ^7^ ^]^ and neuroprosthetics.^[^ ^8^, ^9^ ^]^ The key materials in organic bioelectronics are soft materials that facilitate both electronic and ionic transport, also known as organic mixed ionic‐electronic conductors (OMIECs).^[^ ^10^ ^]^ Among them, the most widely used is PEDOT:PSS,^[^ ^11^ ^]^ an organic material that has been extensively studied and utilized in organic electrodes and organic electrochemical transistors (OECTs).^[^ ^12^ ^]^ The soft nature and mixed conduction of these materials are essential for bioelectronic devices because of two reasons. First, organic mixed conductors enable seamless signal conversion between biological and electronic systems, translating ionic signals into electronic ones. Second, their soft and flexible structure naturally integrates with biological components, such as membranes, due to their similar mechanical properties, ensuring better compatibility and stability.

The key in bioelectronics is their interface, the critical contact point where organic electronic devices interact with biological systems.^[^ ^13^, ^14^ ^]^ The design of such an interface aims to establish a seamless connection between electronic components and biological entities, enabling efficient, multimodal exchange of information and signaling.^[^ ^15^, ^16^, ^17^, ^18^ ^]^ For that reason, biointerfaces that can accommodate and mimic functionally and mechanically the biological environment is of great interest. When focusing on the biology side, cells are surrounded by an external membrane that forms a complex network and, in this way, enables communication between external signals and internal cell components. Developing artificial membranes on top of organic materials that replicate natural functions is therefore essential, as they offer well‐defined platforms for biophysical studies and enable applications ranging from biosensing to bio‐inspired devices and biohybrid integrated circuits.^[^ ^19^, ^20^, ^21^, ^22^ ^]^ One effective approach to achieve this, is through the formation of the Supported Lipid Bilayer (SLB),^[^ ^23^ ^]^ which is a thin lipid bilayer anchored onto a solid substrate. In the simplest form of an interface between an organic mixed conductor and a biocomponent, the transmembrane barrier plays a critical role in regulating the selective exchange of ions and biomolecules and maintaining cellular homeostasis.^[^ ^24^ ^]^ SLBs provide a simplified and versatile platform to model and study the properties of this transmembrane barrier, enabling well‐defined investigations of its permeability and controlled ways to probe it. Combining this simplified membrane model with bioelectronic devices and circuits, important questions have been addressed in the past decade, such as the membrane disruption with toxins,^[^ ^25^ ^]^ the ion channel activity,^[^ ^26^ ^]^ the chemical membrane modification for tailor‐made barrier properties,^[^ ^27^, ^28^ ^]^ and the creation of biohybrid and biomimetic electronics.^[^ ^16^, ^22^, ^29^, ^30^ ^]^ This approach of using a simplified model of membrane offers a stable and well‐defined platform for investigating biological interactions with drugs, toxins, modulators, or even with organic biomimetic electronics.^[^ ^31^, ^32^ ^]^

Despite the obvious advantages of developing well‐controlled biomembrane models such as SLBs, the current methods for producing and integrating SLBs onto bioelectronic or biomimetic devices remain suboptimal, resulting in significant and uncontrolled variations of the transmembrane resistance.^[^ ^24^, ^33^, ^34^ ^]^ Having well‐defined experimental conditions is essential, as they provide numerous advantages such as reliability, reproducibility, precision, and emulation of biorealistic conditions. Before reaching this point, it is essential to understand the sources of membrane variability and to develop simple, analytical assessment methods aimed at designing membranes using well‐defined engineering metrics that go beyond qualitative experimental observations. Recent electrochemical impedance spectroscopy (EIS) studies have provided important insights into the electrochemical behavior and integrity of SLBs on organic substrates, illustrating how impedance spectra reflect bilayer formation and stability.^[^ ^34^ ^]^ However, these analyses typically emphasize phenomenological trends in impedance traces without quantifying the underlying dielectric or structural properties. Therefore, a quantitative framework for understanding transmembrane properties and variability is required, and the definition of physically meaningful descriptors can offer a deeper mechanistic understanding of membrane formation, manipulation, and engineering.

In this work, we investigated the variability of transmembrane resistance in the context of defective membranes, a critical factor influencing the variation, functionality, and stability of bioelectronic interfaces. We developed an analytical model for the equivalent capacitance of the membrane molecular (SLB) stack, which not only describes but also quantifies the observed variations in transmembrane resistance. We define an “SLB stack” as the single‐bilayer head–tail–head dielectric configuration, to clearly distinguish it from multilayer structures. The model shows that the partial hydration of the inner part of the membrane, due to lipid packing non‐idealities and defect formation, leads to variations of the membrane dielectric constant. The membrane dielectric constant is subsequently correlated with the ionic permeability, which thus leads to variations in membrane resistance for nominally the same experimental conditions. Essentially, a higher excess of water in the inner part of the membrane, due to packing defects, shows a qualitative correlation with a decrease in transmembrane resistance. The validity of the model was confirmed in controllable membrane disruption experiments on a single SLB membrane. These findings provide new methodological insights into the dielectric and transmembrane barrier properties of SLBs, enabling a principled and quantitative approach to designing controllable membranes with on‐demand or even biomimetic properties.

## 2 Background

In this work, we focused on the properties and information taken throughEIS regarding the formation of a SLB on top of PEDOT:PSS coated micro array electrodes. Prior to SLB formation vesicles containing a mixture of DOPC and DOTAP in a ratio 4:1 (see Experimental Section for details) were formed as shown in reference.^[^ ^26^ ^]^ Lipid polydispersity, as defined by Dynamic Light Scattering (DLS)s, shows high uniformity of ≈0.1 (monodisperse system), and the vesicle size is found to be 72 ± 6 nm (see Figure S1a, Supporting Information). This uniformity ensures better self‐assembly on top of the PEDOT:PSS film, and therefore enables the formation of a membrane barrier for ion penetration across the membrane. Zeta potential has been calculated for DOPC:DOTAP to be +22 mV ±0.4 (Figure S1c, Supporting Information), which is in agreement with previous studies.^[^ ^34^ ^]^ This value ensures that there is enough stability in order for aggregates not to be formed and that the small addition of DOTAP in the solution provides a higher positive charge in the lipid vesicles. More specifically, a 4:1 molar ratio of DOPC to DOTAP was used to support bilayer formation on PEDOT:PSS surfaces. The inclusion of the cationic lipid DOTAP enhances electrostatic interactions with the negatively charged PSS component, promoting vesicle adsorption and fusion. This formulation also increases the vesicle zeta potential from slightly negative (pure DOPC) to approximately +22 mV, thereby improving colloidal stability and promoting robust membrane adhesion on the polymer surface.

**Figure 1:**
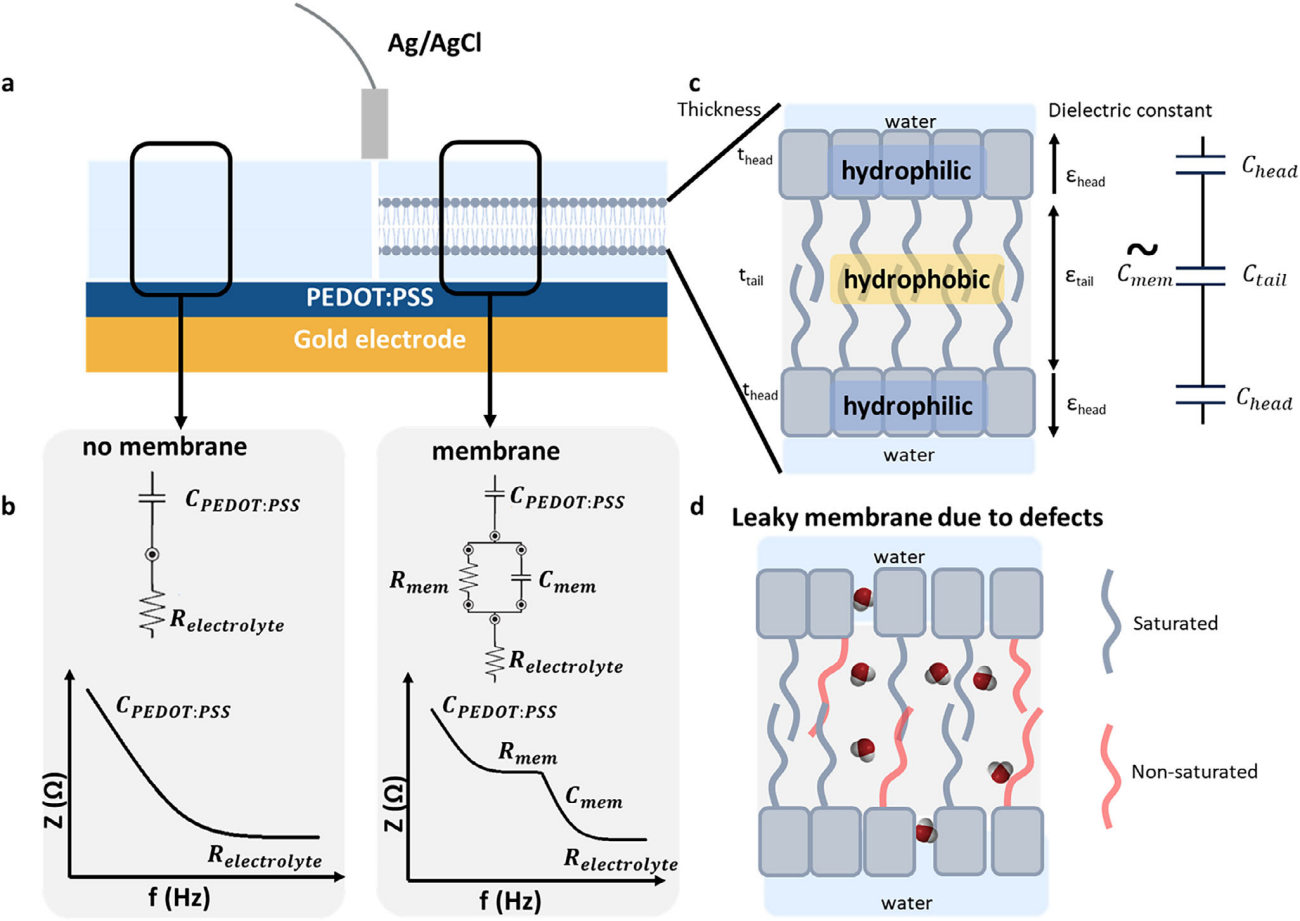
Dielectric and impedance properties of the bilayer stack in ideal and defective membranes. a) Illustration of the studied system of the PEDOT:PSS electrodes in an electrolyte on the left and with the lipid bilayer formation on the right. b) Theoretical impedance spectrum and the corresponding equivalent circuit in the both cases. In case of no membrane there is a 2‐component system, one resistance from the electrolyte (*R_electrolyte_*) and one capacitance of the PEDOT:PSS electrode (*C*_*PEDOT*: *PSS*_). Whereas in the case of a membrane there is a 4‐component system with additional resistance and capacitance of the membrane, *R_mem_* and *C_mem_* respectively. c) Illustration of the bilipid membrane stack and its corresponding sub‐capacitances. The total capacitance from the membrane is portrayed as the *C_mem_*. *C_head_* is the capacitance occurring from the hydrophilic heads of the lipid bilayer and *C_tail_* from the hydrophobic tail. d) Schematic illustration of a leaky membrane due to stacking defects or nanopores.

**Figure** **1**a provides a schematic representation of our biohybrid system. Microarrays consisting of 16 gold electrodes, each measuring 500 µm × 500 µm are coated with PEDOT:PSS. In the first configuration (left panel), the PEDOT:PSS electrode is shown with an electrolyte (PBS) layered on top, serving as the reference setup. The second configuration (right panel) depicts PEDOT:PSS electrodes with SLBs deposited from vesicles, while PBS is also used as the electrolyte.

As shown in Figure 1b the bare PEDOT:PSS gold electrodes can be represented with an equivalent circuit of a series R‐C, meaning a resistance from the electrolyte (*R_electrolyte_*) and a capacitance from the PEDOT:PSS (*C*_*PEDOT*: *PSS*_).^[^ ^35^, ^36^ ^]^ The resistance *R_electrolyte_* expresses the ion conduction mechanism at the bulk of the electrolyte, while *C*_*PEDOT*: *PSS*_ represents the interfacial capacitance of the PEDOT:PSS electrode. In the case of the SLB interfaced PEDOT:PSS electrode, an additional parallel R‐C circuit is added to the previous model, which expresses the membrane resistance (*R_mem_*) and capacitance (*C_mem_*).^[^ ^26^, ^37^, ^38^ ^]^ *R_mem_* accounts for ion penetration across the transmembrane barrier, while *C_mem_* rises because of ionic charge separation across the membrane. Figure 1b also shows a representative EIS spectrum in each of the described cases.^[^ ^35^, ^36^ ^] [^ ^26^, ^37^, ^38^ ^]^

Figure 1c shows schematically the structure of an SLB. A properly formed SLB acts as a barrier to ion penetration, characterized by its membrane resistance *R_mem_*. The separation or accumulation of charge across the membrane is represented by its capacitance *C_mem_*. The membrane comprises two lipid monolayers arranged in a head‐tail‐tail‐head configuration through self‐assembly. The hydrophilic head contains a phosphate group, and the hydrophobic tail is comprised of fatty chains. Due to the difference between head and tail hydrophilicity (or hydrophobicity), this results in the membrane's head‐tail–tail‐head configuration. Therefore, SBLs naturally arrange themselves into a double layer with the hydrophilic heads facing towards the external aqueous environment, while the internal tail–tail region forms a hydrophobic domain, which is depleted from water. The equivalent capacitance *C_mem_* of the head‐tail–tail‐head stack consists of sub‐capacitances which are connected in series:

(1) $$\frac{1}{C_{mem}}=\frac{1}{C_{head}}+\frac{1}{C_{tail}}+\frac{1}{C_{head}}$$

where *C_head_* is the capacitance of each hydrophilic head, facing toward the aqueous phase, and *C_tail_* is the capacitance of the tail–tail hydrophobic domain.

*C_mem_*, *C_head_* and *C_tail_* are given by the following equations:

(2) $$C_{mem}=\varepsilon_{0}\varepsilon_{mem}\frac{A}{t_{mem}}$$

(3) $$C_{head}=\varepsilon_{0}\varepsilon_{head}\frac{A}{t_{head}}$$

(4) $$C_{tail}=\varepsilon_{0}\varepsilon_{tail}\frac{A}{t_{tail}}$$

where ε_*mem*_ is the overall dielectric constant of the membrane (tail‐head‐head‐tail stack), *t_mem_* is the total membrane thickness, *A* its area (which is equal to the electrode area), and ε_0_ the vacuum permittivity. ε_*head*_ is the dielectric constant of the hydrophilic head and *t_head_* is the head thickness. ε_*tail*_ is the overall dielectric constant of the hydrophilic tail–tail domain, and *t_tail_* is the tail–tail thickness.

By combining Equations (1)–(4), we obtain Equation (5), which can be used to determine the dielectric parameters of the membrane stack components (ε_*tail*_ or ε_*head*_), for known geometric parameters (*t_tail_* and *t_head_*).

(5) $$\begin{array}{c} \frac{\varepsilon_{\mathrm{mem}}}{t_{\mathrm{mem}}}=\frac{\varepsilon_{\mathrm{head}}\cdot \varepsilon_{\mathrm{tail}}}{2\cdot \varepsilon_{\mathrm{tail}}\cdot t_{\mathrm{head}}+\varepsilon_{\mathrm{head}}\cdot t_{\mathrm{tail}}}=\frac{\varepsilon_{\mathrm{head}}\cdot \varepsilon_{\mathrm{tail}}}{\varepsilon_{\mathrm{tail}}\cdot t_{\mathrm{mem}}+t_{\mathrm{tail}}\left(\varepsilon_{\mathrm{head}}-\varepsilon_{\mathrm{tail}}\right)}\;\;\; \end{array}$$

A more detailed schematic, also considering the chemical structure of SBLs is shown in (Figure S2, Supporting Information). The total thickness *t_mem_* of the membrane is *t_mem_* = 4.3 nm (determined experimentally using ellipsometry, Figure S4, Supporting Information),^[^ ^39^ ^]^ the head thickness *t_head_* = 1 nm (defined from its chemical structure) and the tail–tail thickness *t_tail_* = *t_mem_*‐2 · *t_head_* = 2.3 nm.

Since the head group is terminating towards the external aqueous environment, the dielectric constant of the head compartment is regarded to be equal to that of water, ε_*head*_ = 78.4. Ideally, in a hydrophobic environment that is fully depleted from water, the dielectric constant of the tail–tail stack is that of typical hydrocarbons, ε_*tail*_ = 2.2.^[^ ^40^, ^41^ ^]^ However, real biological membranes as well as bilipid membranes depart from this ideal model and usually form stacking defects between the lipids or even nanovoids though the membrane (Figure 1d).^[^ ^42^ ^]^ This non‐ideal stacking of the lipids or the nanovoid formation may lead to water‐rich pathways for ion penetration within the membrane and increase the ionic leakage through it. In order to capture this non‐ideality, an effective dielectric constant of the tail is defined, ε_*tail*,*eff*_, which takes into account the partial hydration of the hydrophobic tail–tail stack in the presence of defects or nanovoids. Essentially, assuming a homogenous distribution of water molecules in the tail–tail domain, ε_*tail*,*eff*_ equals partially to the dielectric constant of water and the dielectric constant of the remaining dry tail–tail domain.

(6) $$\varepsilon_{tail,eff}=V_{H2O}\cdot \varepsilon_{H2O}+\left(1-V_{H2O}\right)\cdot \varepsilon_{tail}$$

where ε_*H*2*O*_ = 78.4 is the dielectric constant of water occupying a volume fraction *V*_*H*2*O*_ in the tail–tail domain, ε_*tail*_ = 2.2^[^ ^40^, ^41^ ^]^ is the dielectric constant of the dry tail occupying the remaining content (1‐V_H2O_). Calculating the ε_*tail*,*eff*_ then gives an indication of the water volume fraction still remaining inside the tail (resulting in ε_*tail*,*eff*_>2.2) due to packing defects and/or nanovoids. In the limiting case of “dry” tail–tail domain, *V_H2O_* = 0 and ε_*tail*,*eff*_ = 2.2 equals to that of hydrocarbons.

## 3 Results

**Figure 2:**
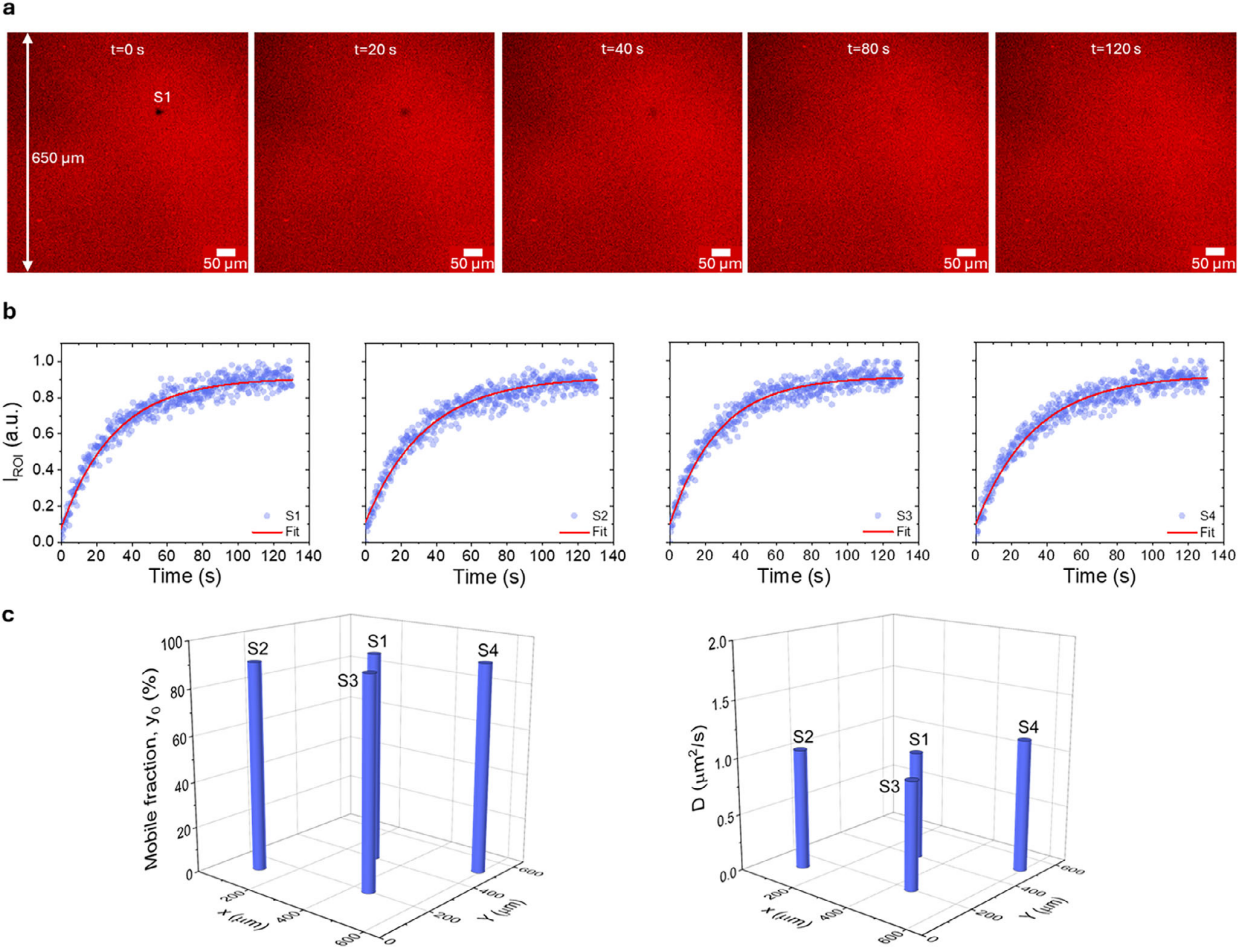
Validity of the model: Probing the membrane homogeneity and single SLB kinetics. a) Fluorescence recovery after photobleaching (FRAP) measurements of SLB on a PEDOT:PSS film with an area of 650 µm × 650 µm, at a specific spot (S1). Frames correspond to different time points during fluorescence recovery (t = 0–120 s). b) Normalized Fluorescence Intensity recovery, I_ROI_ versus Time, for four different positions S1‐S4 sampled across the area of 650 µm × 650 µm. Exponential fits to the recovery curves are also shown. c) Extracted mobile fraction of SLBs *y_0_*, and diffusion coefficient *D*, for the four different positions S1‐S4 across the sampled area of 650 µm × 650 µm. spatial sampling indicates that the majority of SLBs are mobile (y_0_> 90%) with diffusion coefficient D≈1 µm2/s, consistent with literature values. The image of fluorescence recovery, as well as both *y_0_* and *D* are uniform across the sampled area and in a biophysical range of values, supporting the presence of a single homogeneous SLB.

The validity of the model relies on two key assumptions: that a single SLB is formed and that it provides homogeneous coverage of the electrode surface. To address this, we performed fluorescence recovery after photobleaching (FRAP) measurements at different positions within a 650 µm × 650 µm region of an SLB supported on a PEDOT:PSS film prepared under the same conditions as the electrodes (**Figure** **2**). This area is comparable to the typical electrode area used for electrochemical characterization (500 µm × 500 µm). Four random positions (S1–S4) were probed with a bleach spot radius of w = 10 µm. A representative FRAP experiment at S1 is shown in Figure 2a. Even by visual inspection, the recovery images appear homogeneous (apart from the bleached area), suggesting uniform presence of fluorophores and thus homogeneous membrane coverage. Figure 2b presents the normalized fluorescence recovery curves (I_ROI_ vs Time) for the four positions, which were analyzed using a single‐exponential function, $I_{ROI}=y_{0}+A_{0}e^{-t/t_{R}})$, with *I_ROI_* the Normalized Fluorescence Intensity, *y_0_* the mobile fraction of the area with radius *w* after photobleaching, *t_R_* the recovery constant.^[^ ^43^, ^44^ ^]^ When the post‐bleach baseline approaches unity (*y_0_* →1), essentially all fluorophores are mobile, indicating the presence of a continuous SLB rather than multilayer stacks. In contrast, additional biomaterial atop an SLB would hinder lipid mobility, yielding a significant residual intensity (*y_0_* << 1). The membrane diffusion coefficient was extracted according to $D=3.23\frac{W^{2}}{t_{R}}$.^[^ ^43^ ^]^ As shown in Figure 2c, all probed spots exhibited mobile fractions above 90%, indicating the absence of significant immobile residuals and thus providing strong evidence for the formation of a single SLB across the area. Moreover, the measured diffusion coefficients of D≈1 µm2/s are consistent with literature values for single SLBs (0.5–2 µm^2^/s).^[^ ^23^, ^45^, ^46^ ^]^ In contrast, multilayer SLBs typically exhibit lower apparent mobility and significant immobile fractions, which is inconsistent with the present observations. Overall, the FRAP data obtained and shown in Figure 2 confirm that the SLB, in the form of a single layer, is homogeneous across the sampled region and support the validity of our model. Based on the model validation, the presence of a single SLB allows the use of its experimental thickness (here t_mem_ = 4.3 nm), in the analysis with relative confidence. It should be noted that validation of the SLB layer under study is essential and must be established prior to any formal analysis, in order to ensure the applicability of the model.

**Figure 3:**
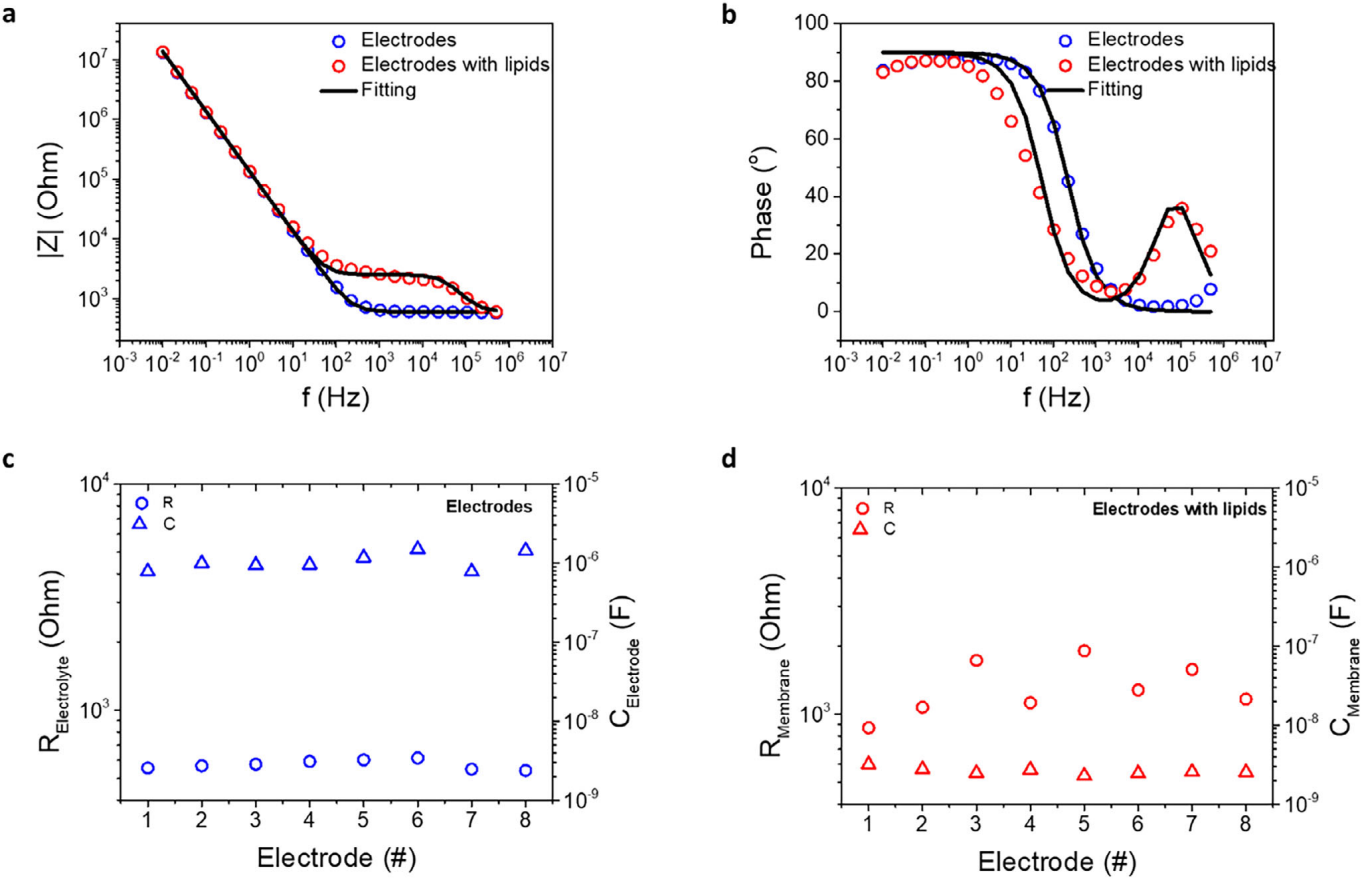
Statistical analysis of membrane‐to‐membrane variation. a) EIS spectrum (magnitude vs frequency) of PEDOT:PPS electrodes and PEDOT:PPS electrodes with lipids including the corresponding equivalent circuit modelling. b) EIS spectrum (phase vs frequency) of the PEDOT:PPS electrodes and the electrodes with lipids including the corresponding equivalent circuit modelling. c) Extracted parameters from equivalent circuit modelling (electrolyte resistance *R_electrolyte_* and electrode capacitance *C*_*PEDOT*: *PSS*_) for N = 8 measured electrodes for nominally the same experimental conditions. d) Extracted parameters from equivalent circuit modelling (membrane resistance *R_mem_* and membrane capacitance *C_mem_*) for N = 8 measured electrodes for nominally the same experimental conditions.

The EIS spectrum of the PEDOT:PSS electrodes was measured in plain electrodes with PBS (before the addition of the lipid solution), and after forming the SLBs (**Figure** **3**). Figure 3a shows the impedance amplitude versus frequency spectrum for a PEDOT:PSS electrode (in PBS) and with lipids, while in Figure 3b the phase versus frequency spectrum is plotted. PEDOT:PSS electrodes show typical EIS characteristics, either in the magnitude or phase spectrum, while in the case of lipids, an intermediate regime is appearing, which is the characteristic signature of a transmembrane barrier.^[^ ^25^, ^34^ ^]^ Fitting the EIS spectrum with the equivalent circuit models of Figure 1b is also shown. Moreover, the same EIS spectrum is measured for nominally the same conditions, i.e., on the same substrate, with the same treatment and on the same day, for 8 electrodes in total in the case of PEDOT:PSS electrodes without and with lipids. The corresponding Bode plot diagrams of all electrodes are shown in detail in Figure S3 (Supporting Information).

Figure 3c,d shows the equivalent circuit parameters as extracted from EIS fitting for PEDOT:PSS electrodes and PEDOT:PSS electrodes with lipids, respectively. Initially, the EIS fitting was performed for the electrodes in order to extract the electrolyte resistance *R_electrolyte_* and the electrode capacitance *C*_*PEDOT*: *PSS*_. As a next step, the lipids were deposited on the very same electrodes (for more details, see Experimental Section) and the previous *R_electrolyte_* and *C*_*PEDOT*: *PSS*_ parameter values of each PEDOT:PSS electrode were used for extracting the membrane resistance *R_mem_* and membrane capacitance *C_mem_* for each electrode. In the case of PEDOT:PSS electrodes, *R_electrolyte_* and *C*_*PEDOT*: *PSS*_ show a relatively small fluctuation, with *R_electrolyte_* = 575 ± 4% Ohm and *C*_*PEDOT*: *PSS*_ = 1.06 ± 21% µF. The volumetric capacitance C* of PEDOT:PSS, as extracted from the mean electrode capacitance of all electrodes, is in agreement with previous works, C* = 36±21% F cm^−3^.^[^ ^47^, ^48^ ^]^ In the case of PEDOT:PSS electrodes with lipids, *R_mem_* = 1340 ± 27% Ohm and *C_mem_* = 2.62 ± 12% nF. The addition of lipids does not cause significant variations in capacitance fluctuation (i.e, 12% for bare electrodes and 21% for bilayer‐interfaced electrodes). However, the transmembrane resistance fluctuates significantly with respect to the reference electrolyte resistance (from 4% to 27%). These observations are consistent with the equivalent circuit behavior of heterogeneous membranes, where local resistive and capacitive contributions add in parallel. Specifically, the total resistance *R_eq_* follows $\frac{1}{R_{eq}}=\sum_{i}\frac{1}{R_{i}}$, and is therefore dominated by the lowest local resistances such as those at defect sites, leading to large overall changes in conductance. In contrast, the total capacitance $C_{eq}=\sum_{i}C_{i}$ increases linearly with each local contribution, making the effect of small, high‐dielectric defects relatively minor unless their areal density is significant. It should be noted that the quality of impedance spectroscopy fitting can be slightly improved by using power‐law elements such as a constant‐phase element (CPE), which account for non‐idealities such as parameter distributions (e.g., resistance, capacitance), frequency‐dependent behavior, or the presence of defects.^[^ ^49^ ^]^ Nevertheless, the simple equivalent circuit captures the essential behavior (Figure S6, Supporting Information).

It should be mentioned that based on the overall experiments in this work, typical areal values for membrane resistance and capacitance are *R_mem_* = 4–12.5 Ohm∙cm^2^ and C_mem_ = 1.0–1.2 µF cm^−2^, while typical values for defect‐free membranes are *R_mem_* = 1–10 mOhm∙cm^2^ and C_mem_ = 1.0 µF cm^−2^.^[^ ^50^ ^]^ The measured capacitance values (C_mem_ = 1.0–1.2 µF cm^−2^) fall within the expected range for fluid SLBs and are slightly higher than typical literature values, which can be attributed to slightly polar membranes due to water. However, the resistance range (*R_mem_* = 4–12.5 Ohm∙cm^2^) is significantly lower than this of defect‐free membranes. Taken together, the near‐canonical capacitance plus uniform and physically relevant FRAP kinetics of Figure 2 (y_0_> 90%, D≈1 µm2/s) support a single, continuous SLB on PEDOT:PSS. The low areal resistance indicates it is leaky, likely due to parallel defects/pathways rather than multilayer stacks or patchy membranes. To further clarify the relationship between membrane coverage and electrical properties, it is important to note that the combination of a capacitance value characteristic of a complete bilayer and a comparatively low resistance is diagnostic of a “continuous but leaky” SLB. In contrast, incomplete or patchy coverage would lead to a substantial reduction in the effective areal capacitance and a dominant contribution from the underlying electrode, inconsistent with the experimental data. The results therefore support the presence of a laterally continuous bilayer that maintains overall structural integrity, while localized defects or partial hydration pathways contribute to the observed ionic leakage.

This difference in fluctuation for nominally the same lipid membranes is further investigated in the context of the partial hydration of the tail–tail domain as a result of the non‐ideal lipid packing. We hypothesize that this membrane resistance variability for seemingly the same membranes, is caused by lipid packing non‐idealities, which enable the ionic transport via hydration channels across the membrane (Figure 1d). As it will be shown, this partial hydration of the membrane impacts the dielectric properties of the tail‐to‐tail domain.

**Figure 4:**
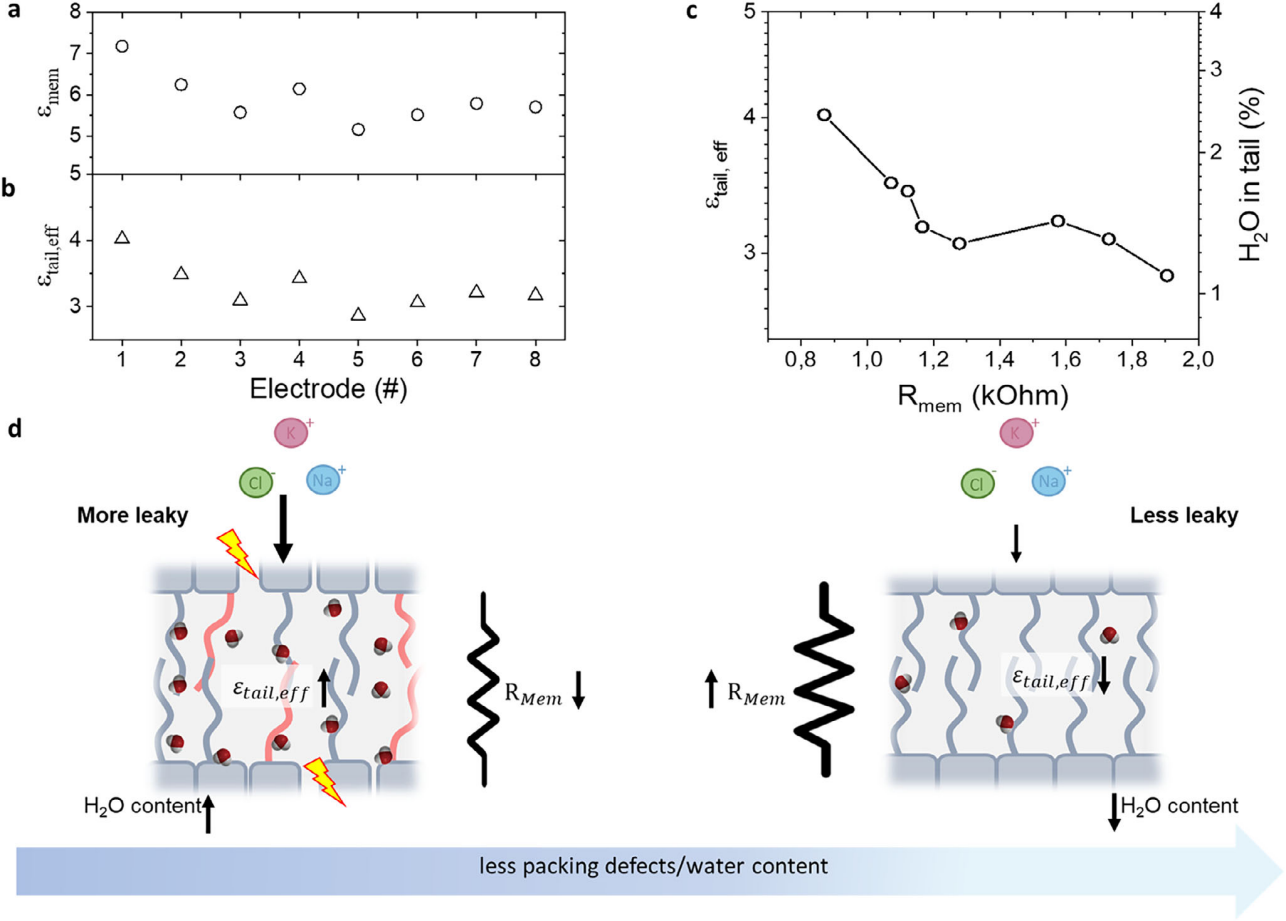
Associating membrane variations to their dielectric properties. a) Dielectric constant of the membrane ε_*mem*_ versus the number of electrodes. b) Effective dielectric constant of the tail–tail ε_*tail*,*eff*_ versus the number electrodes (N = 8, nominally the same membrane) showing the different values occurred in each electrode. c) ε_*tail*,*eff*_ along with the water content in the tail for each corresponding *R_mem_* (nominally the same membrane). d) Mechanistic description of the transmembrane resistance variability. Membranes with higher (areal) density of packing defects, display higher effective dielectric constant, ε_*tail*,*eff*_, at the tail–tail compartment due to the presence of water molecules. Water molecules in hydrated (leaky) membranes enable transmembrane ion transport via water channels, and this results in a decreased transmembrane resistance, *R_mem_*_._

**Figure** **4** provides a mechanistic scenario for the variations in membrane resistance as a result of packing defects and can be deciphered by studying the dielectric properties of the membrane. In detail, Figure 4a shows the dielectric constant of the membrane stack (head‐tail‐tail‐head) ε_*mem*_ for each SBL (nominally same *N* membranes, N = 8), as calculated from the membrane capacitance *C_mem_* of each EIS for nominally the same membranes. ε_*mem*_ values for each SBL vary from 5.16 to 7.18. Equations (2)–(5) are used to define the dielectric constant for the tail ε_*tail*_ (ε_*tail*_>2.2, so ε_*tail*_ is regarded as ε_*tail*,*eff*_) for each SBL for known thicknesses (*t_mem_*, *t_tail_* and *t_head_*) and dielectric constants (ε_*mem*_ and ε_*head*_). As shown in Figure 4b, ε_*tail*,*eff*_ varies from 2.86 to 4.02 (ε_*tail*,*eff*_ = 3.28 ± 12%). In order to understand this fluctuation for nominally the same SBLs and associate it with physical and experimentally accessible parameters, Figure 4c shows a correlation of the transmembrane resistance of each SBL with the tail effective dielectric constant, ε_*tail*,*eff*_. As expected, ε_*tail*,*eff*_ is always higher (> 2.2) than that of “dry” hydrocarbons (2.2), and most importantly it follows an inversely proportional relationship with the transmembrane resistance, *R_mem_*. Assuming a partial hydration of the membrane due to non‐idealities of lipid packing, Equation (6) is used to calculate the water content for each membrane (1.0–2.5% water).

Figure 4c proves the scenario of partial membrane hydration and how this impacts the transmembrane resistance for nominally the same SBLs (with *R_mem_* = 1340 ± 27% Ohm). This is schematically shown in Figure 4d for two representative cases of low and high *R_mem_*. In the case of a “leaky” membrane, non‐idealities of lipid packing promote water diffusion at the inner part of the membrane, and this is reflected as a higher effective dielectric constant of the tail–tail compartment, ε_*tail*,*eff*_, as ε_*H*2*O*_>>ε_*tail*_. In such defect‐mediated hydrated membranes, water channels form pathways for transmembrane ion conduction and this consequently translates in a low transmembrane barrier, or *R_mem_*. In the opposite direction, membranes with less defects and better lipid packing, impede water diffusion inside the tail–tail compartment and therefore the formation of transmembrane water channels. This is reflected as a lower ε_*tail*,*eff*_, lower water content and subsequently as a higher barrier/resistance for transmembrane ion transport *R_mem_*.

**Figure 5:**
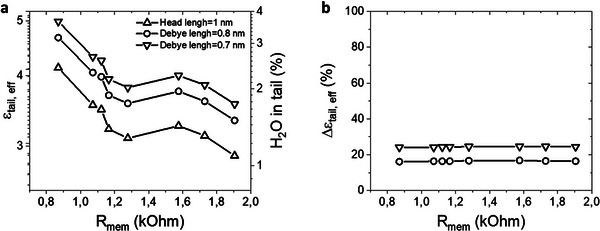
Impact of the head capacitance. a) Effective dielectric constant of the tail, *ε_tail,eff_* as a function of the membrane resistance *R_mem_* for different thickness of the head capacitor. *t_head_* = 1 nm corresponds to the length of the molecular head group. *t_head_* = 0.8 nm when the thickness of the head capacitor is regarded as the Debye length of the PBS solution with a dielectric of water, *ε_r_* = 78.4. *t_head_* = 0.7 nm when the thickness of the head capacitor is regarded as the Debye length of the PBS solution with a dielectric constant of lipid heads in aqueous environment, *ε_r_* =30. b) Percentage change of *ε_tail,eff_*, Δ*ε_tail,eff_* for the modified *t_head_* values in respect to the molecular length of the head (*t_head_* = 1 nm) as a function of the membrane resistance *R_mem_* for different thickness of the head capacitor.

It should be noted that, in order to simplify the model and isolate the essential features, we made two baseline assumptions regarding the headgroup capacitance, *C_head_*, and, as a consequence regarding the behavior of Figure 4. First, we assumed that the dielectric constant of the headgroup region is equivalent to that of bulk water, *ε_head_* = 78.4. Second, we approximated the thickness of this region as the molecular length of the DOPC headgroup, *t_head_* = 1 nm. However, a more detailed treatment of *C_head_* is necessary to be evaluated, by approximating the thickness of the head capacitor with the Debye length *λ_D_* in PBS. More specifically, the ionic composition of PBS is 137 mm NaCl, 2.7 mm KCl, 10 mm Na_2_HPO_4_ (2Na⁺ + HPO_4_^2−^), 1.8 mm KH_2_PO_4_ (K⁺ + H_2_PO_4_^−^). The ionic strength of PBS is $I_{PBS}=\frac{1}{2}\sum c_{i}z_{i}^{2}$, where *c_i_* the molar concentration and *z_i_* the charge of ion *i*. By taking into account the composition of PBS, its ionic strength is *I_PBS_* = 0.17 m. Assuming that the head capacitance is approximated with the Debye length *λ_D_* in PBS, the modified thickness of the head capacitance is $t_{head}^{\prime }$ = $\lambda_{D}=\sqrt{\frac{\varepsilon_{r}\varepsilon_{0}K_{B}T}{2N_{A}e^{2}I_{PBS}}}$, where *ε_r_* is the dielectric constant of the medium, *ε_0_* the vacuum permittivity, *K_B_* the Boltzmann constant, *T* the temperature, *N_A_* the Avogadro's number, *e* the elementary charge. By taking into account the above modifications, we have calculated the Debye length λ_*D*_ (or $t_{head}^{\prime }$), based on the value of *I_PBS_* in the case of the dielectric constant of the medium *ε_r_* for water (*ε_r_* = 78.4) and by using a mean value of the dielectric constant of lipid heads in an aqueous environment (*ε_r_* = 30).^[^ ^51^ ^]^ This results in a modified thickness of the head with values 0.8 and 0.7 nm, respectively. **Figure** **5** shows the effective dielectric constant of the tail, *ε_tail,eff_* as a function of the membrane resistance *R_mem_*. The modified values of the *t_head_* lead to a small and systematic increase of *ε_tail,eff_* as the new *t_head_* is now lower, and small corrections are introduced with the modified *t_head_* (≈16% and ≈24% increase for *t_head_* = 0.8 nm and *t_head_* = 0.7 nm, respectively). It should be noted that the simplified model still captures the essential experimental behavior, while a more detailed description of the headgroup region provides additional refinements.

**Figure 6:**
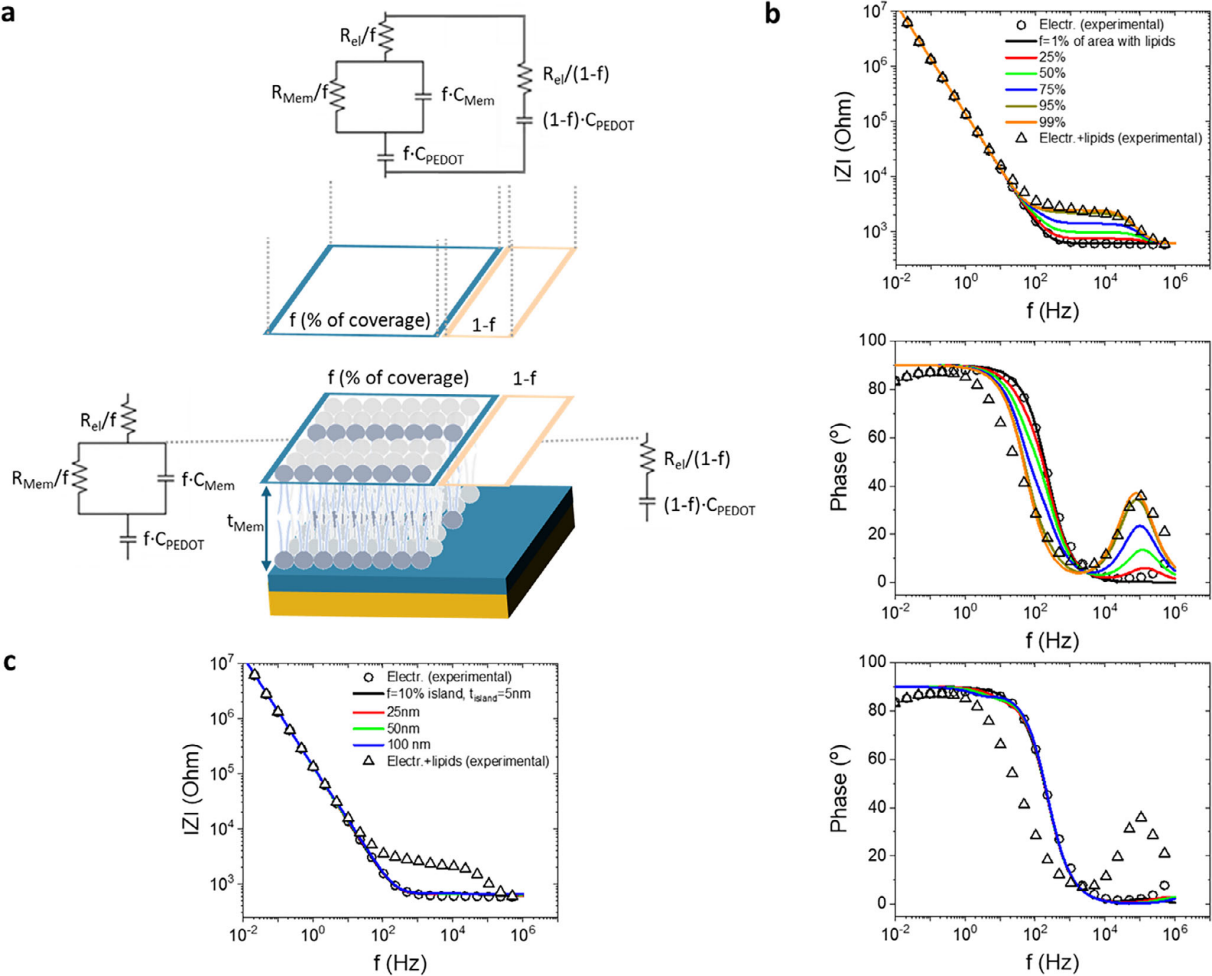
Membrane non‐idealities. a) Equivalent circuit of a PEDOT:PSS electrode that is partially covered with SBL, with area fraction *f* and thickness *t_Mem_*. The remaining PEDOT:PSS electrode has an area fraction of (1‐*f*). b) Simulations of the IS spectrum (Amplitude and Phase) for different factional SBL coverage *f* = 1–99% using the equivalent circuit of Figure 6a. Experimental spectra for bare PEDOT:PSS electrode and nominally covered electrode with SBL are also shown for reference. Parameters used for simulations: *R_El_* = 603.5 Ohm, *R_mem_* = 1905 Ohm, *C_Mem_* = 2.288 × 10^−9^ F, *C_PEDOT_* = 1.171 × 10^−6^ F, *A* = 500 × 500 µm2. c) Simulations of the IS spectrum (Amplitude and Phase) for an lipid island with coverage *f* = 10% and different thicknesses, t_island_ = 5‐100 nm. Experimental spectra for bare PEDOT:PSS electrode and nominally covered electrode with SBL are also shown for reference. Parameters used for simulations: *R_El_* = 603.5 Ohm, *R_mem_* = 1905·(*t_island_*/5 nm) Ohm, *C_Mem_* = 2.288 × 10^−9^·(5 nm/*t_island_*) F, *C_PEDOT_* = 1.171 × 10^−6^ F, *A* = 500 × 500 µm2.

It is also important to consider non‐idealities such as the possibility of partial electrode coverage with lipids. In this analysis, a fraction *f* of the electrode is covered by the SLB is assumed, while the remaining fraction (1−*f*) corresponds to bare PEDOT:PSS (**Figure** **6**). This scenario results in two parallel equivalent circuits: one representing the PEDOT:PSS electrode covered by the SLB (area *A* fraction *f*), and one representing the bare PEDOT:PSS electrode (area fraction 1−*f*). Individual circuit elements scale with *f* as indicated in Figure 6a. Figure 6b shows the simulations of the IS spectrum (Amplitude and Phase) for different fractional SBL coverage *f* = 1–99% using the equivalent circuit of Figure 6a. Practically, *f* ranges from bare electrode (*f* = 1%) to fully covered electrode (*f* = 99%). The experimental spectra for bare PEDOT:PSS electrode and the nominally covered electrode are also shown for reference. The simulations show that only fractions approaching full coverage, f> 95%, are sufficient to reproduce the experimental behavior. As an additional confirmation, for f ≈ 0%, the simulated spectrum reproduces the experimental one of the bare PEDOT:PSS electrode. The possibility of partial electrode coverage is even further considered, and the effect of a membrane island with different thickness *t_island_* is studied. A fraction *f* = 10% of the electrode is covered by the SLB, effectively simulating the formation of a membrane island. Figure 6c shows the simulations of the IS spectrum (Amplitude and Phase) for membrane islands with different thicknesses (*t_island_* = 5–100 nm). Practically, *t_island_* ranges from the nominal membrane thickness value (*t_island_* = 5) to a high value which represents a “multi‐SLB island” (*t_island_* = 100). The simulations suggest that in the case of isolated islands (i.e., fractional coverage of the total electrode size with variable thickness), the Amplitude and Phase remain practically the same as the spectrum is dominated by the impedance PEDOT:PSS bare electrode. Therefore, in the case of islands (e.g., small electrode coverage), the of exposed PEDOT:PSS will dominate the EIS regardless of the lipid thickness. These results further support the scenario that the electrode is homogeneously covered with a relatively leaky membrane.

It should be mentioned that the nanoroughness of the PEDOT:PSS surface may also play a role in the observed bilayer properties. Unlike atomically flat substrates such as mica, conducting polymer surfaces exhibit intrinsic nanoscale topography, which can influence vesicle fusion dynamics, membrane continuity, and ionic transport pathways across the bilayer. Such effects could partially contribute to the relatively low resistance values observed here (*R_mem_* = 4–12.5 Ohm∙cm^2^), despite the confirmed continuous coverage. Recent studies have shown that substrate roughness can significantly affect the formation and stability of SLBs, underscoring its importance in the design of bioelectronic interfaces.^[^ ^52^ ^]^

**Figure 7:**
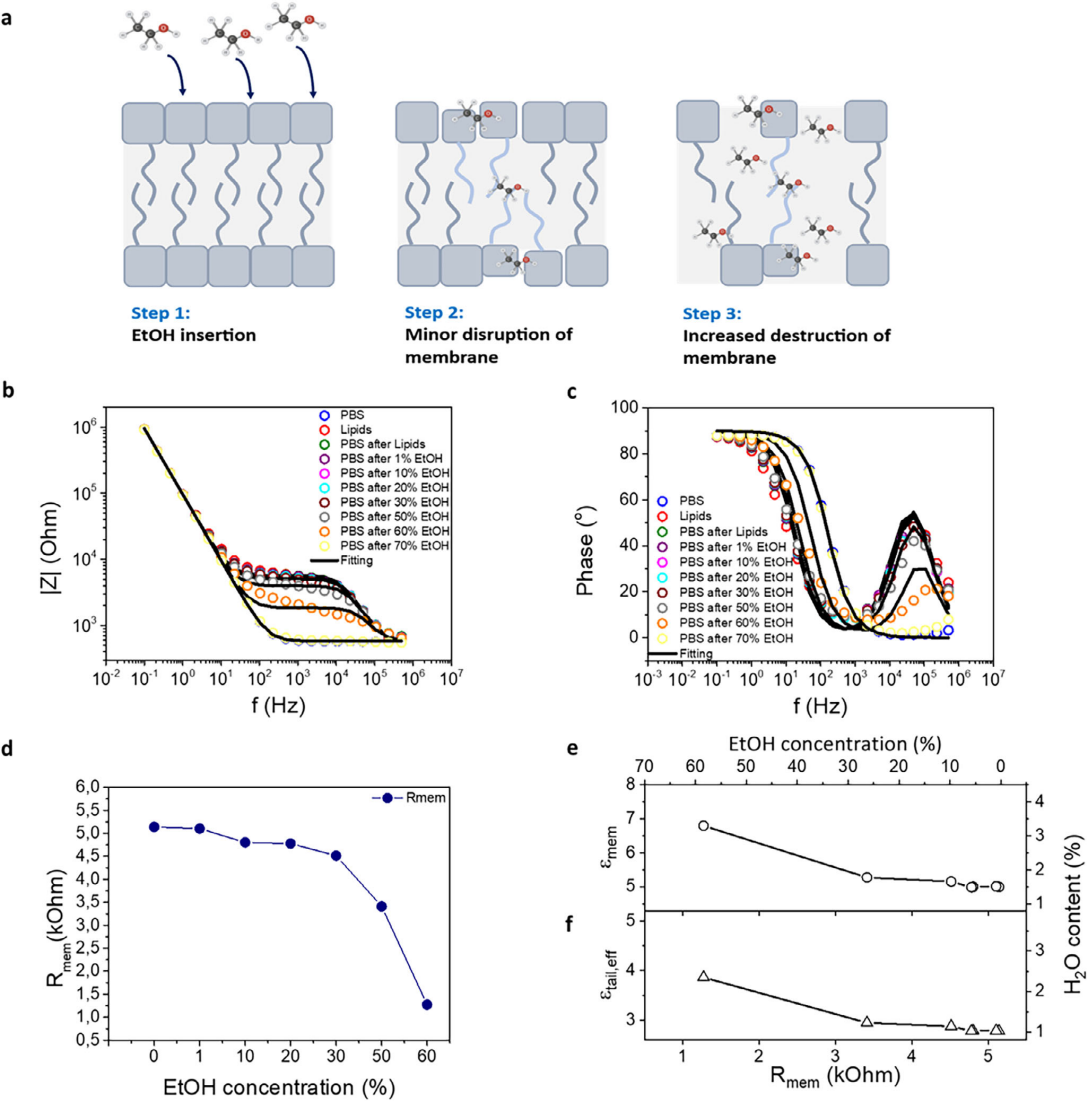
Verification of the simplified model with controlled addition of EtOH in the system. a) Schematic mechanism showing the slow destruction of the SLB with the addition of EtOH. b,c) Representation of Bode plot before and after the SLB is formed, and as a function of EtOH concentration. When the membrane is destroyed the measurements overlap with the baseline. d) *R_mem_* for each concentration of EtOH showing gradual membrane disruption. e) Dielectric constant of the membrane ε_*mem*_ and water content in the tail versus *R_mem_* and EtOH concentration. f) Effective dielectric constant of the tail ε_*tail*,*eff*_and water content in the tail versus *R_mem_* and EtOH concentration.

To verify the validity of the simple membrane dielectric description, a study was performed in a well‐defined context where the SLB is disrupted using ethanol, a known membrane disruptor reference.^[^ ^26^ ^]^ For this purpose, EtOH was added, in a controlled manner, then washed by PBS, and measured with EIS. Specifically, the EtOH concentration was progressively increased until the SLB was fully disrupted, thus recovering the original and the electrode‐only spectrum (no SLB formation). The mechanism of this procedure is shown in **Figure** **7**a, where EtOH firstly starts to insert the membrane (step 1), later it starts disrupting the lipid formation (step 2) and finally it manages to destroy the membrane completely (step 3). Figure 7b,c shows a representative Bode plot with the changes in the SLB while adding the EtOH. The EIS spectra and equivalent circuit fitting from each condition and concentration are shown in Figure S5 (Supporting Information). Figure 7d shows the change in the *R_mem_* as a function of EtOH concentration. It is observed that after adding 60% EtOH the *R_mem_* drops to almost half than the intact lipid bilayer. After adding 70% EtOH, there is no SLB anymore and the curve perfectly overlaps with that previously measured without SLB (PBS only).

Further analysis was done using the dielectric model of the membrane, correlating the dielectric properties of the membrane with the EtOH concentration, the *R_mem_*, and the corresponding water content of the inner part of the membrane. In this experiment, the use of EtOH provides a controllable access of *R_mem_*, and therefore a well‐defined correlation between *R_mem_* and water content. In agreement with the experiment displayed in Figure 4, lower *R_mem_* (probed with higher EtOH concentrations) leads to higher water content in the inner part of the membrane. This verifies the validity of the dielectric description of the SLB membrane. It should be noted the presence of charged lipids (addition of a small ratio DOTAP in DOPC) does not alter the central analysis, as the focus remains on the relative electrical properties of the membrane, specifically, changes in resistance and effective dielectric constant under controlled lipid composition and deposition conditions.

## 4 Conclusion

Most studies on the formation of SLBs as synthetic model membranes on organic mixed conductor devices, such as electrodes and OECTs, with a common denominator the membrane‐to‐membrane transmembrane variability. Here, we explored this variability of transmembrane resistance by developing an analytical model for the equivalent membrane capacitance of the molecular head‐tail‐head stack (i.e., an SLB). We first assessed the applicability of our model by probing spatiotemporally the kinetics of the membrane. Our findings indicate that lipid packing imperfections lead to partial hydration within the membrane's inner region, altering its dielectric constant. This change in dielectric properties directly correlates with ionic permeability, resulting in transmembrane resistance fluctuations under otherwise identical experimental conditions. We validated our model through controlled membrane disruption experiments on a SLB. In this study, we employ a streamlined impedance spectroscopy approach to quantify membrane properties through physically meaningful metrics or even with simple fractional descriptors of impedance contributions. In this way, effective metrics derived from impedance can be leveraged for engineering SLBs and their sub‐compartments, beyond the simple evaluation of SLB formation, in a manner similar to how device engineers use extracted parameters to understand and optimize device performance. Rather than relying solely on qualitative interpretation of the impedance spectrum, parameters such as the dielectric constant of the tail region and the estimated water content are extracted to serve as benchmarks for the design and optimization of new, multifunctional or multicomponent membranes on top of conducting polymer films. For instance, while the raw impedance spectra may initially appear to reflect random experimental variability, analysis of the dielectric properties reveals a consistent and interpretable pattern across samples. Therefore, the results offer a methodology and framework that transcend qualitative experimental observations for engineering more predictable, tuneable, and multifunctional biomimetic membranes on top of biosensors or bio‐inspired devices.

## 5 Experimental Section

### Electrode Fabrication

The electrodes were fabricated using microfabrication techniques. The substrates used were standard microscope glass slides of dimensions 25 × 75 mm. These substrates were first cleaned in a sonicated bath by using a soap solution (Micro‐90 (Sigma–Aldrich)) and then in a solution 1:1 of Acetone and Isopropanol. These were used to create electrodes by depositing photolithographically patterned gold with the aid of positive Microposit S1813 photoresist (DOW). 5 nm layer of chromium was evaporated under the gold to help with the adhesion. Two layers of parylene C were deposited with the addition of soap in between to facilitate the removal of the top parylene C layer. In addition, silane A‐174 (γ‐methacryloxypropyl trimethoxysilane) (Sigma–Aldrich) was incorporated into the first parylene C layer to improve adhesion. The dimensions of the electrodes were defined in the second photolithography step using the positive photoresist AZ‐10XT MicroChemicals (Cipec Spécialités). Reactive ion etching (O_2_ plasma, 200 W for 14 min with an O_2_ flow rate of 50 sccm) was then employed to etch the electrodes through the photoresist mask. On each glass substrate, there were 16 electrodes of 200 µm × 200 µm and another 16 electrodes of 500 µm × 500 µm dimensions. The mixed electronic‐ion conductor polymer used in this work was PEDOT:PSS (Clevios PH 1000) mixed with 5.0 wt.% ethylene glycol, 0.1 wt.% dodecyl benzene sulfonic acid, and 1.0 wt.% (3‐glycidyloxypropyl)trimethoxysilane. Spin coating was used to produce a film in two steps at 1000 rpm and 3000 rpm for 1 min, and annealed at 120 °C for 1 min in between and resulting in a nominal thickness of ≈300–400 nm. After the peel‐off of the upper parylene C layer, a hard‐baking step was applied at 140 °C for 60 min.

### Liposome Preparation

1,2‐dioleoyl‐sn‐glycero‐3‐phosphocholine (DOPC) and 1,2‐dioleoyl‐3‐trimethylammonium‐propane (DOTAP) (Avanti Polar Lipids) solutions in chloroform were combined at a 4:1 ratio. This mixture was dried under nitrogen gas, followed by vacuum drying at room temperature for one hour to remove any remaining chloroform. The dried lipids were then rehydrated in PBS to achieve a concentration of 4 mg mL^−1^. This lipid suspension was frozen at −20 °C overnight, and subsequently extruded ≈20 times through a 100 nm polycarbonate membrane (GE Healthcare). After that, the solution was examined with Dynamic Light Scattering (DLS) for its polydispersity (PDI) as well as its lipid vesicle size. Zeta potential measurements were also performed for further characterization of the lipid vesicles and to ensure the stability of the DOPC:DOTAP in PBS. DOPC in PBS was also checked as a reference and compared the results with the literature.

### Preparation of Supported Lipid Bilayers (SLBs)

300 µL of liposomes (DOPC:DOTAP 4 mg mL^−1^ in PBS) were added by vesicle fusion technique on PEDOT:PSS coated electrodes. Prior to the lipid deposition, the PEDOT:PSS film was treated with O_2_ plasma for 120 s. In order to improve the surface roughness and activate it. The liposomes were incubated for 20–30 min and measured by EIS. A polycarbonyl well was glued to create a stable environment for the lipid incubation in the microelectrode area.

### Electrochemical Impedance Spectroscopy (EIS)

A potentiostat was used for EIS measurements (Palm Sens4 by Palm Sens), recording impedance spectra in the frequency range between 0.01 Hz and 500 kHz with a sampling density of 3 frequency points/decade. It should be noted that increasing the sampling density to 5 points/decade did not substantially alter the overall fitting quality or the extracted parameters. The measurements were performed by connecting the potentiostat to a probe station and using the Pt electrode as counter electrode, and Ag/AgCl pellet electrode was the reference electrode, and the working electrode was a microfabricated gold PEDOT:PSS electrode (plain or with SLBs). For acquiring the EIS spectrum, an AC voltage with an amplitude of 0.2 V and a DC voltage of 0 V were applied. All measurements were recorded in 300 µL PBS or with the lipid solution (retained on the electrode area by a polycarbonyl glued well).

### Elipsometry

Spectroscopic ellipsometry measurements were performed on DOPC:DOTAP SLBs deposited on silica wafers using a Quantum Design spectroscopic ellipsometer. Data were acquired at three incidence angles (65°, 70°, and 75°) and analysed using the CompleteEASE software package. The optical model consisted of a silicon substrate with a native SiO_2_ layer and a homogeneous lipid overlayer. To accurately determine the SLB thickness, the bare silica wafer was measured first to determine the oxide layer thickness, which was then subtracted from the total fitted thickness. The lipid layer was modelled using a “Cauchy film” model, which is an empirical optical model describing the wavelength‐dependent refractive index of transparent thin films with negligible absorption, commonly used to extract thicknesses of organic layers such as lipid bilayers. The effective refractive index of 1,44 for DOPC and 1,33 for DOTAP were assigned, consistent with hydrated DOPC:DOTAP bilayers in PBS.

## Conflict of Interest

The authors declare no conflict of interest.

## Supporting information



Supporting Information

## Data Availability

The data that support the findings of this study are available from the corresponding author upon reasonable request.
