# Supplementary material for: A Methodology for Deciphering the Transmembrane Resistance Variability of Supported Lipid Bilayers
Source: Adv Sci (Weinh). 2025 Nov 10;13(6):e08589. doi: 10.1002/advs.202508589 (PMC12866879; doi:10.1002/advs.202508589)
Supplement: Supplementary file 1 — Supporting Information [file ADVS-13-e08589-s001.docx]

Supporting Information

Deciphering the transmembrane resistance variability of supported lipid bilayers

Aristea Pavlou, Debdatta Panigrahi, Anna-Maria Pappa, Fabrizio Torricelli, Paul W.M. Blom, Paschalis Gkoupidenis *

Correspondence to P.G. ([gkoupidenis@mpip-mainz.mpg.de](mailto:gkoupidenis@mpip-mainz.mpg.de) , [pgkoupi@ncsu.edu](mailto:pgkoupi@ncsu.edu) )


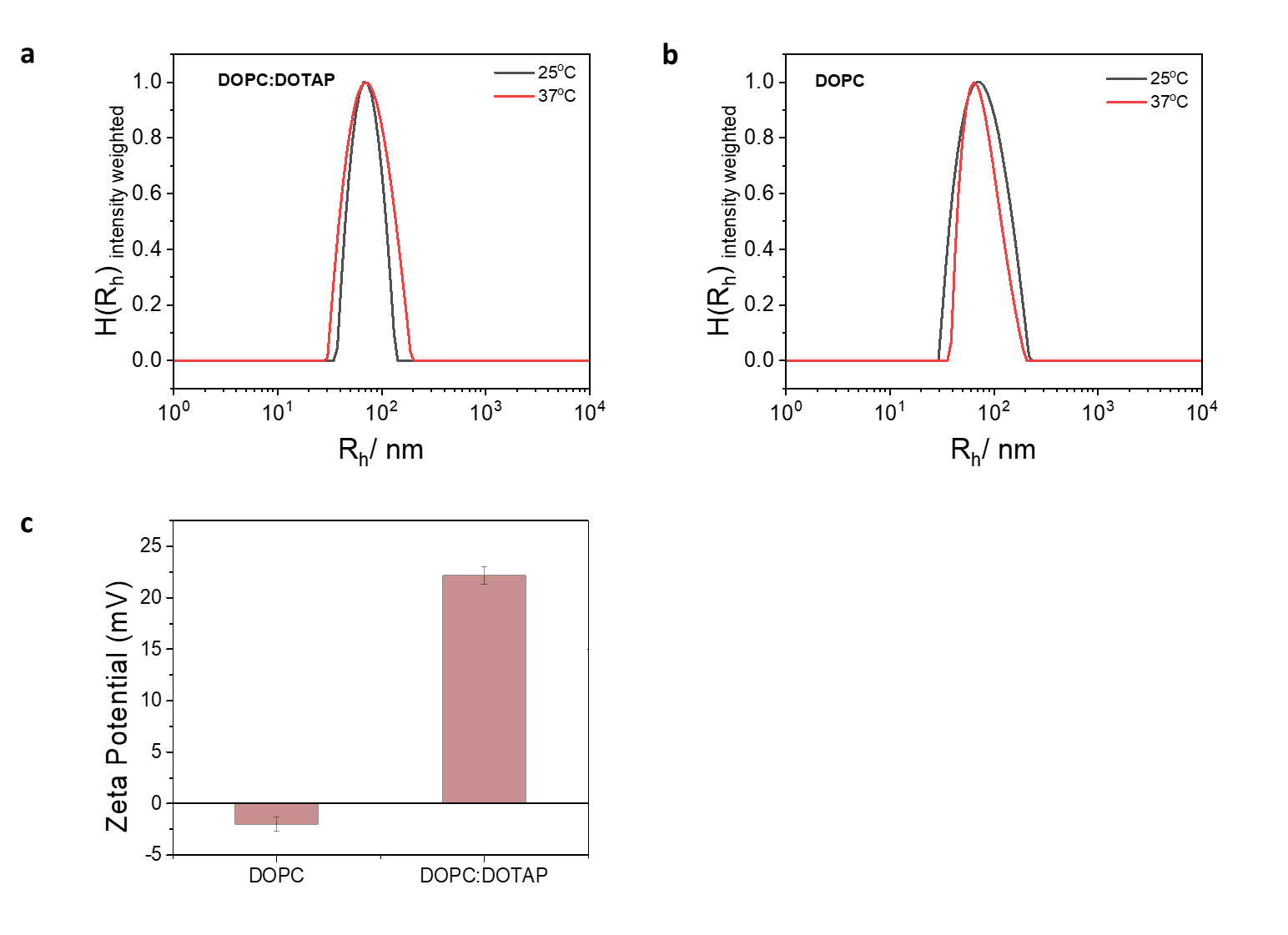


***Figure S1:*** Liposome size and zeta potential characterization by Dynamic Light Scattering (DLS). **(a)** Size characterization in both room and body temperature for the extruded solutions of DOPC:DOTAP in a ratio of 4:1 and **(b)**DOPC showing that the size is approximately 75nm for DOPC:DOTAP and 82nm for DOPC in both temperatures, **(c)** Zeta-potential of both DOPC alone and DOPC:DOTAP mixture, where the value for DOPC is around -2mV and for DOPC:DOTAP is +22mV as also reported in the literature.^[53]^


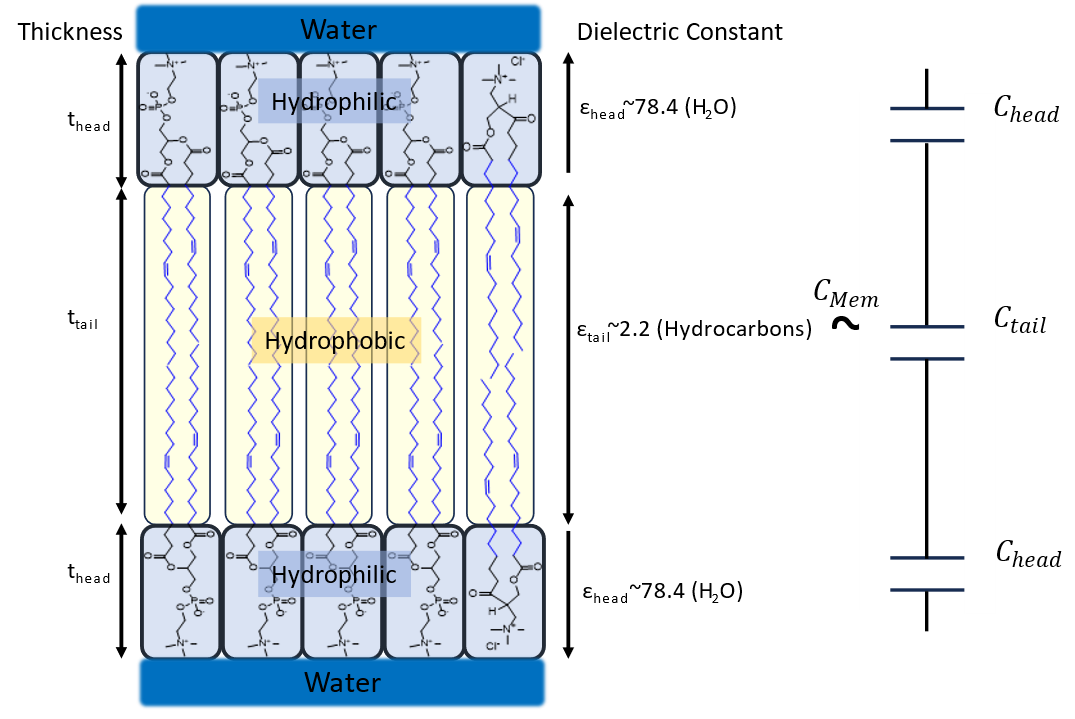
**Figure S2:** Illustration of SLBs with chemical structures of DOPC:DOTAP (4:1). DOPC is shown in the first four bilayer columns on the left and the last one on the right is refered to the DOTAP.


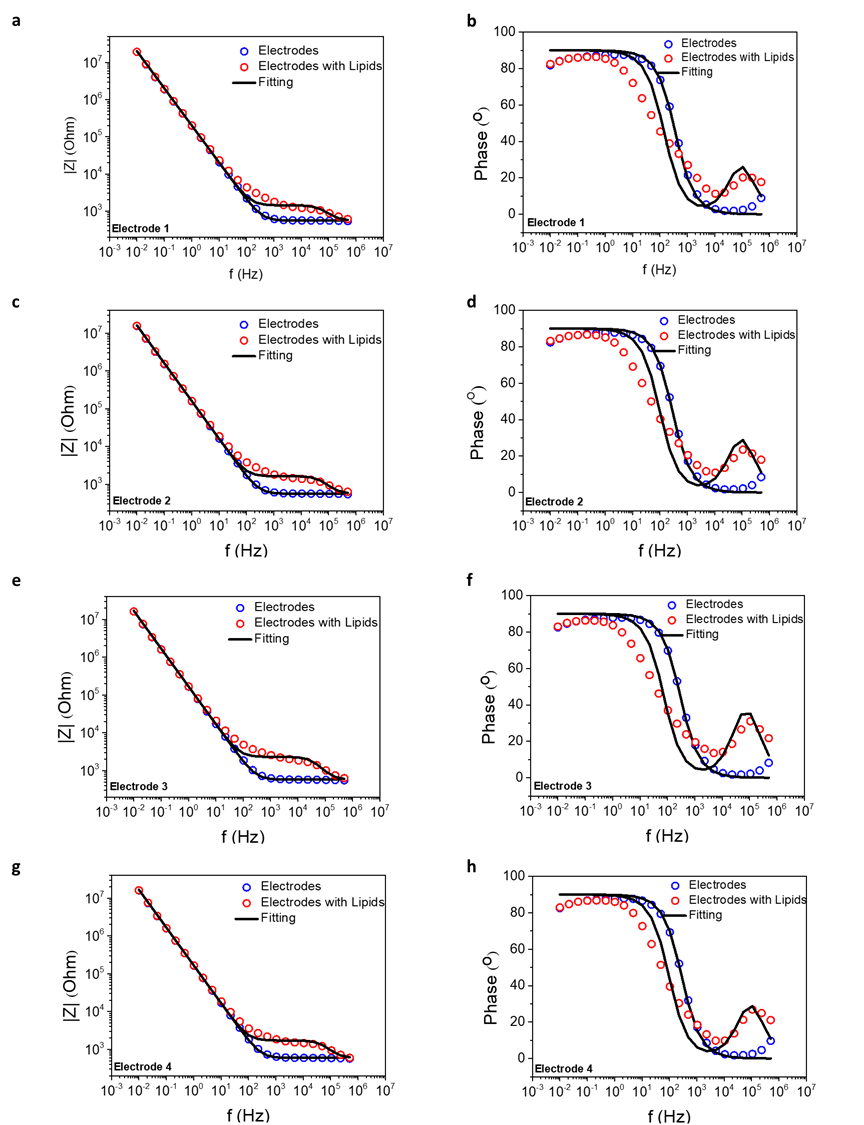


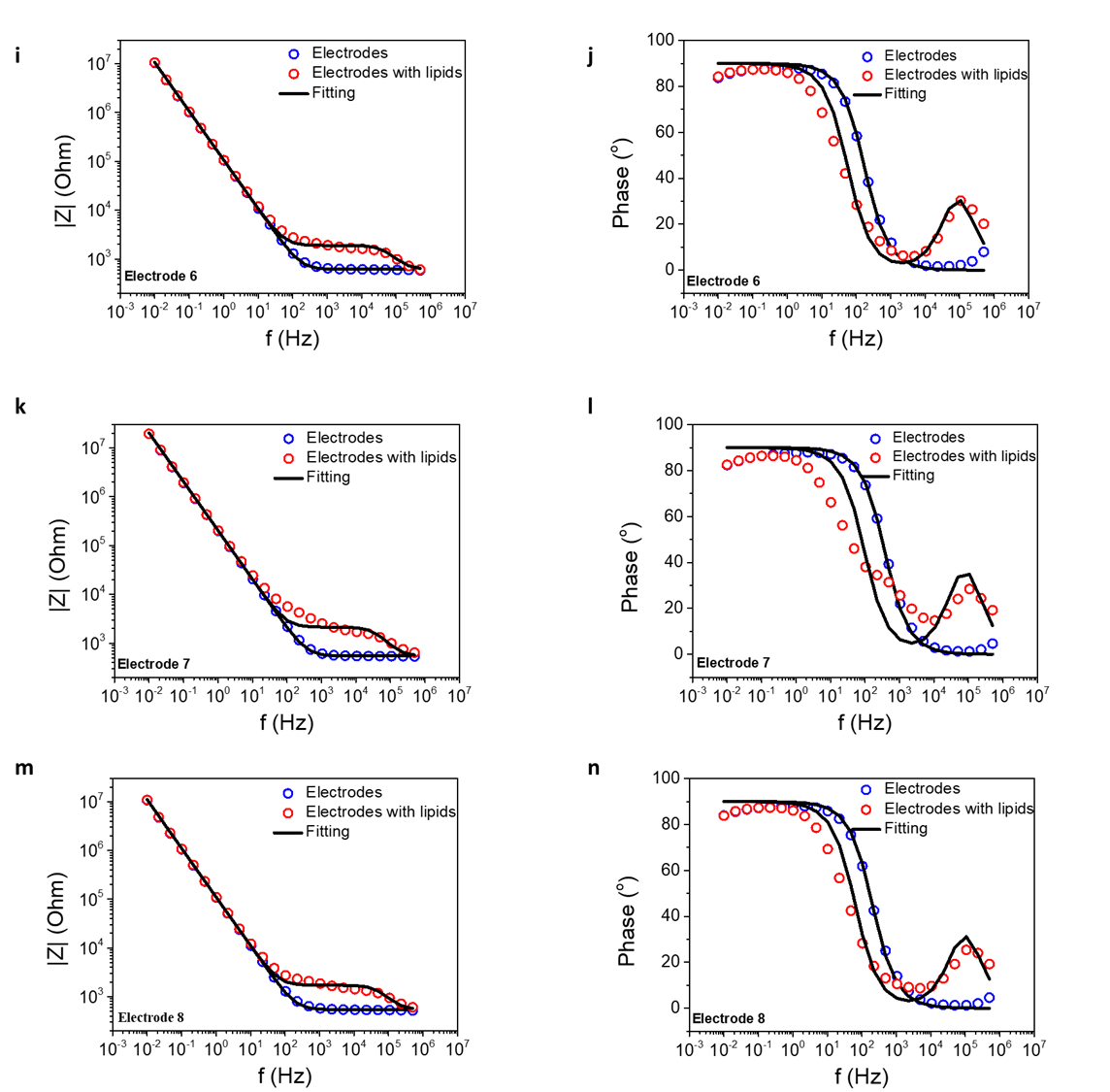


**Figure S3:** Representation of the of the Bode plots showing the EIS spectra from each electrode (N=7) measured before and after the formation of the lipid membrane. Electrode 5 is shown in the Fig. 2.

**
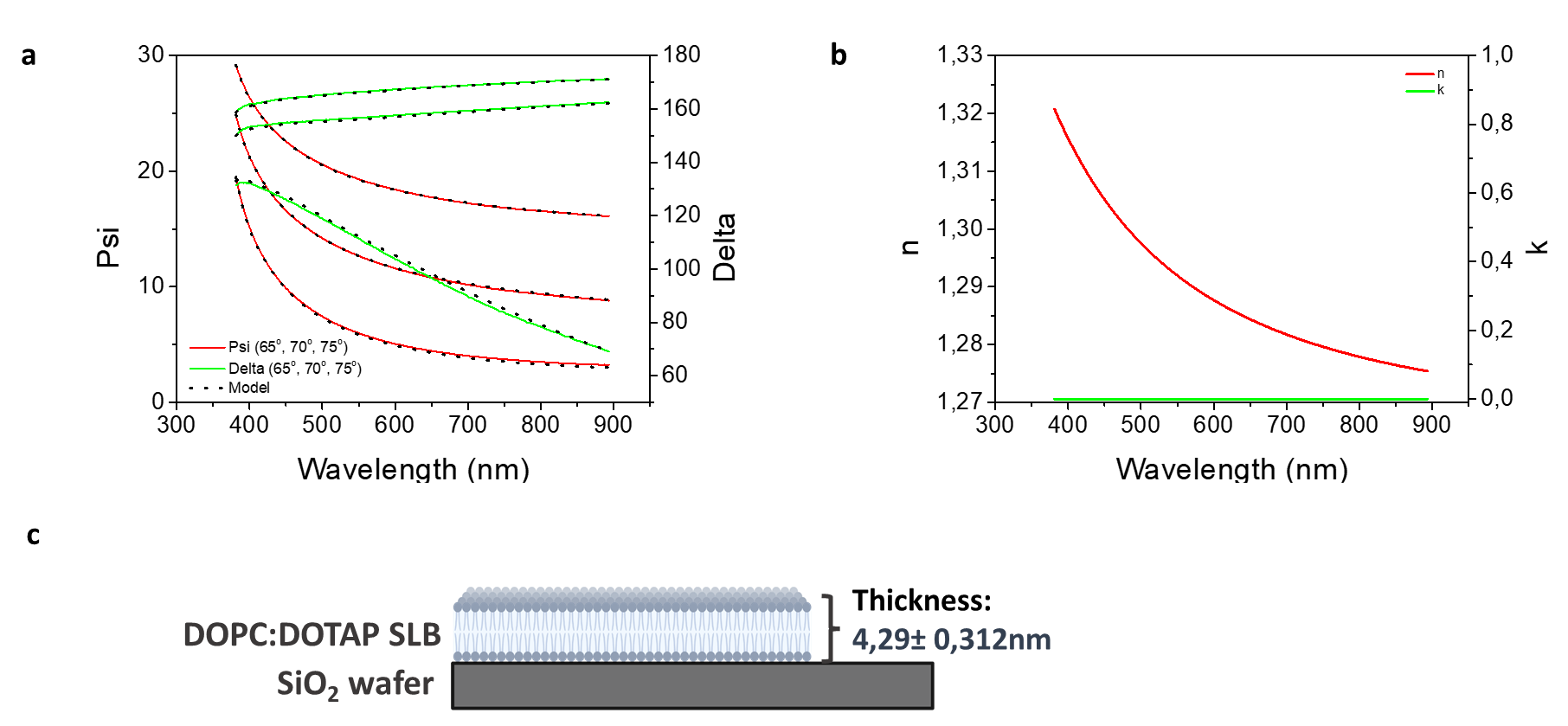
**

**Figure S4:** Spectroscopic Ellipsometry results on silica wafer with DOPC:DOTAP SLB performed by Quantum Design Spectroscopic Ellipsometer on 3 different angles (65^o^,70^o^,75^o^) analyzed by CompleteEASE software using the “Cauchy film” model. **(a)** Diagram of Psi and Delta values vs the wavelength measured in the above mentioned 3 angles and fitted with the “Cauchy film” model. **(b)** Diagram of the measured n (refractive index) and k (extinction coefficient) values vs the wavelength , where the n and k values are in accordance with the expected ones for DOPC:DOTAP properties, **(c)** Schematic representation of the SLB on silica wafer portraying the measured thickness.


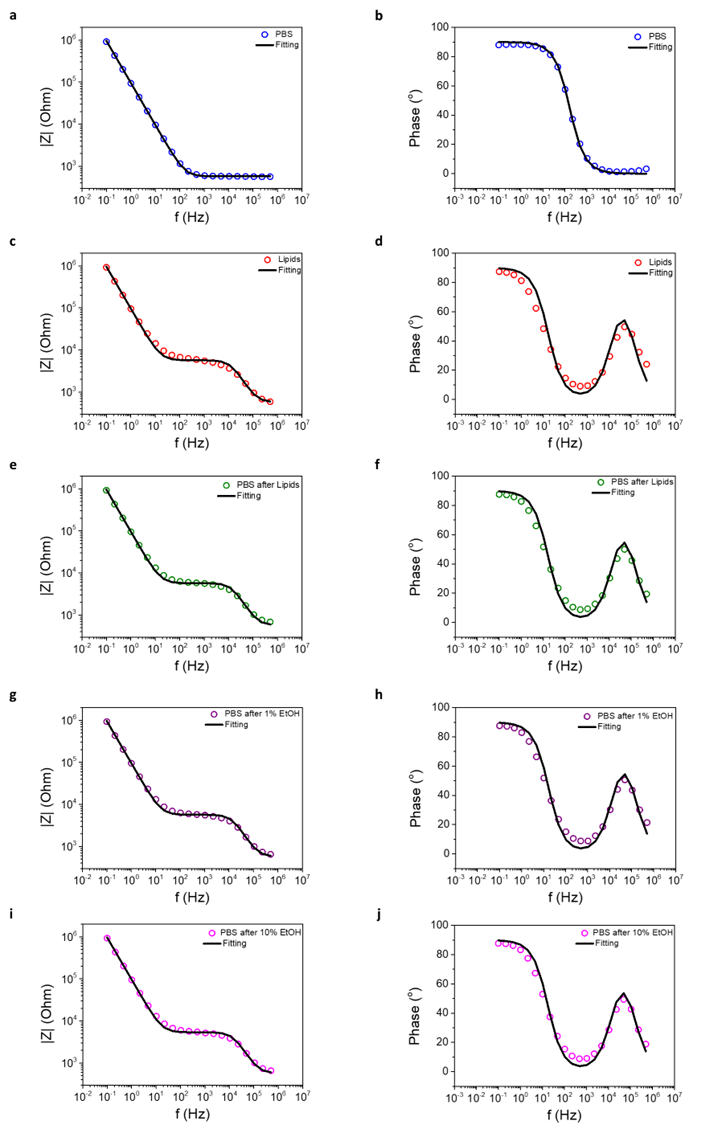

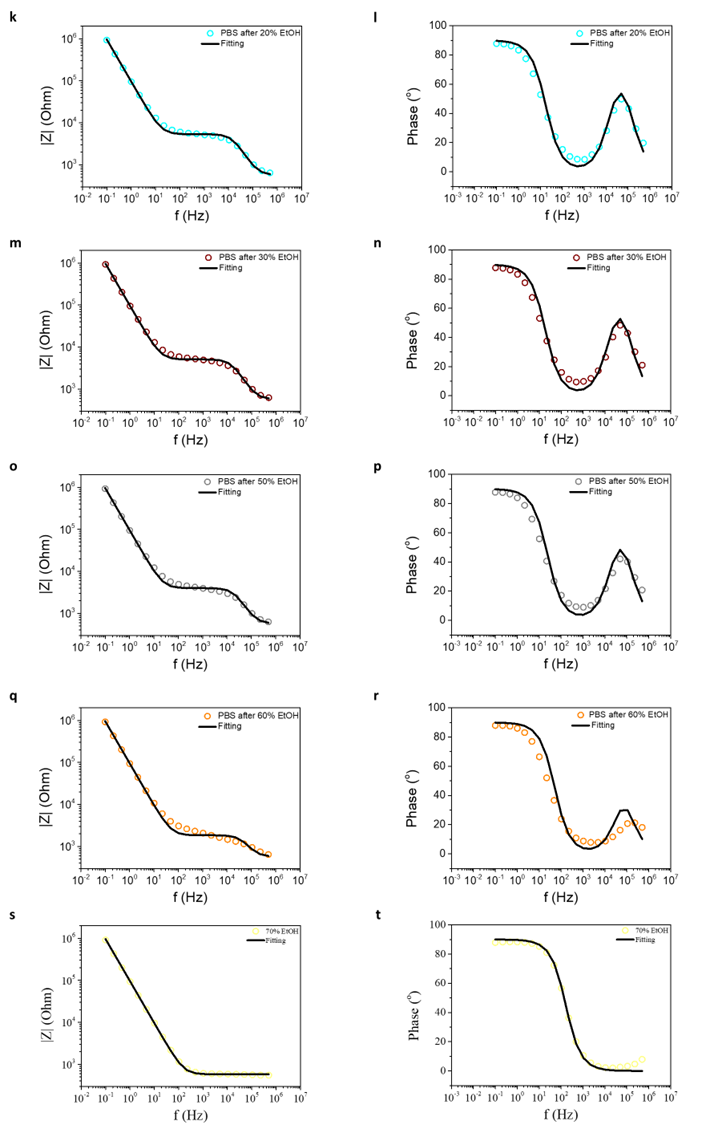


**Figure S5:** Separate plots of the different conditions and EtOH concentrations from the moment of the gold PEDOT:PSS electrodes with PBS to the formation of the SLB and the addition of each concentration of EtOH until the complete destruction of the SLB.


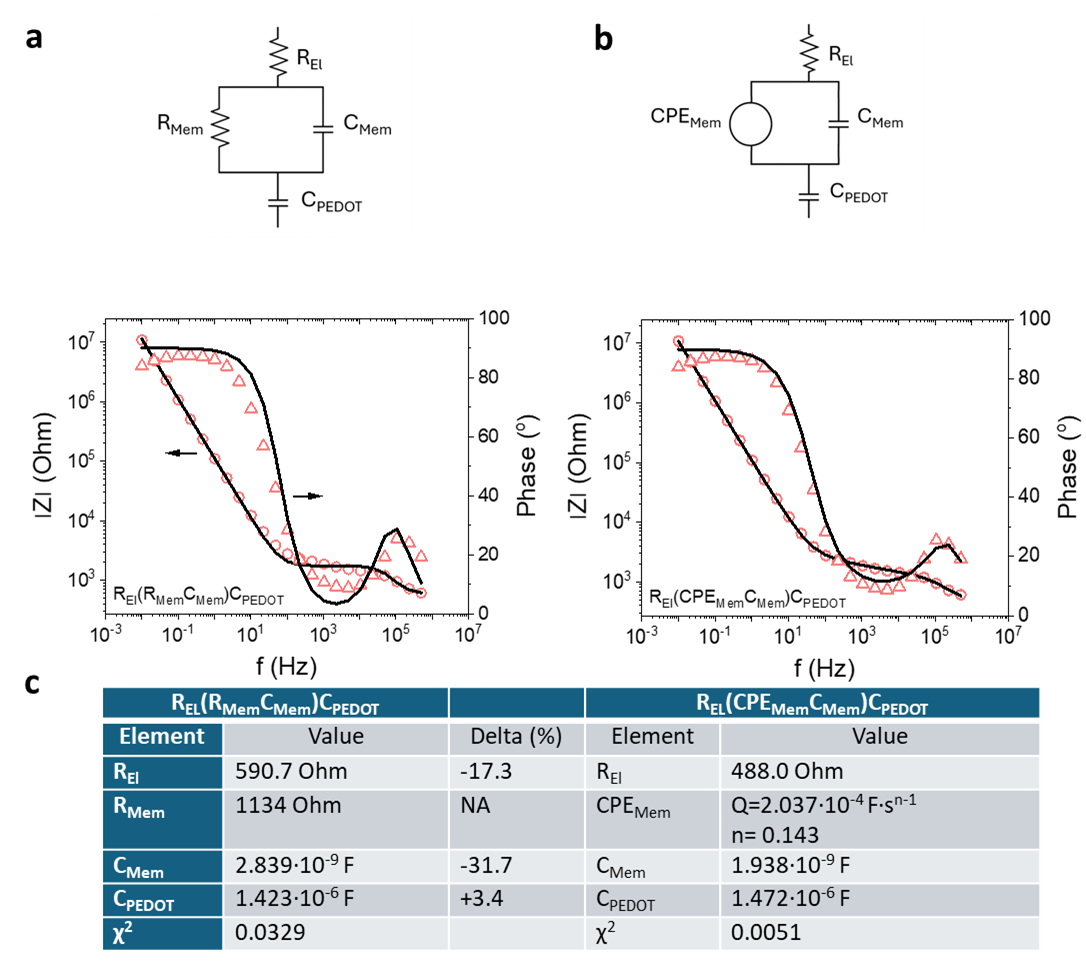


**Figure S6:** Equivalent circuit modelling of a representative electrode with SBLs for: **(a)** R_EL_(R_Mem_C_Mem_)C_PEDOT_, **(b)** R_EL_(CPE_Mem_C_Mem_)C_PEDOT_. **(c)** Table with fitting parameters for both circuits. The quality of the of fitting is improved when using a Constant Phase Element (CPE) element.

**References**

[1] C. Pitsalidis, A. M. Pappa, A. J. Boys, Y. Fu, C. M. Moysidou, D. van Niekerk, J. Saez, A. Savva, D. Iandolo, R. M. Owens, *Chem. Rev.* **2022**, *122*, 4700.

[2] J. Rivnay, R. M. Owens, G. G. Malliaras, *Chem. Mater.* **2014**, *26*, 679.

[3] G. Malliaras, I. McCulloch, *Chem. Rev.* **2022**, *122*, 4323.

[4] A. Marks, S. Griggs, N. Gasparini, M. Moser, *Adv. Mater. Interfaces* **2022**, *9*, 1.

[5] D. T. Simon, S. Kurup, K. C. Larsson, R. Hori, K. Tybrandt, M. Goiny, E. W. H. Jager, M. Berggren, B. Canlon, A. Richter-Dahlfors, *Nat. Mater.* **2009**, *8*, 742.

[6] A. Kalmykov, C. Huang, J. Bliley, D. Shiwarski, J. Tashman, A. Abdullah, S. K. Rastogi, S. Shukla, E. Mataev, A. W. Feinberg, K. Jimmy Hsia, T. Cohen-Karni, *Sci. Adv.* **2019**, *5*, DOI 10.1126/sciadv.aax0729.

[7] D. Khodagholy, T. Doublet, P. Quilichini, M. Gurfinkel, P. Leleux, A. Ghestem, E. Ismailova, T. Hervé, S. Sanaur, C. Bernard, G. G. Malliaras, *Nat. Commun.* **2013**, *4*, DOI 10.1038/ncomms2573.

[8] F. Iberite, J. Muheim, O. Akouissi, S. Gallo, G. Rognini, F. Morosato, A. Clerc, M. Kalff, E. Gruppioni, S. Micera, S. Shokur, *Science (80-. ).* **2023**, *380*, 731.

[9] I. Krauhausen, S. Griggs, I. McCulloch, J. M. J. den Toonder, P. Gkoupidenis, Y. van de Burgt, *Nat. Commun.* **2024**, *15*, 1.

[10] B. D. Paulsen, K. Tybrandt, E. Stavrinidou, J. Rivnay, *Nat. Mater.* **2020**, *19*, 13.

[11] C. Boehler, Z. Aqrawe, M. Asplund, *Bioelectron. Med.* **2019**, *2*, 89.

[12] S. L. Bidinger, S. Han, G. G. Malliaras, T. Hasan, *Appl. Phys. Lett.* **2022**, *120*, DOI 10.1063/5.0079011.

[13] M. Fahlman, S. Fabiano, V. Gueskine, D. Simon, M. Berggren, X. Crispin, *Nat. Rev. Mater.* **2019**, *4*, 627.

[14] A. Saleh, A. Koklu, I. Uguz, A.-M. Pappa, S. Inal, *Nat. Rev. Bioeng.* **2024**, *2*, 559.

[15] C. Lubrano, G. M. Matrone, C. Forro, Z. Jahed, A. Offenhaeusser, A. Salleo, B. Cui, F. Santoro, *MRS Commun.* **2020**, *10*, 398.

[16] P. Gkoupidenis, Y. Zhang, H. Kleemann, H. Ling, F. Santoro, S. Fabiano, A. Salleo, Y. van de Burgt, *Nat. Rev. Mater.* **2024**, *9*, 134.

[17] M. J. Donahue, A. Sanchez-Sanchez, S. Inal, J. Qu, R. M. Owens, D. Mecerreyes, G. G. Malliaras, D. C. Martin, *Mater. Sci. Eng. R Reports* **2020**, *140*, 100546.

[18] X. Strakosas, M. Bongo, R. M. Owens, *J. Appl. Polym. Sci.* **2015**, *132*, 1.

[19] Z. Lu, C. Barberio, A. Fernandez‐Villegas, A. Withers, A. Wheeler, K. Kallitsis, E. Martinelli, A. Savva, B. M. Hess, A. Pappa, G. S. K. Schierle, R. M. Owens, *Adv. Sci.* **2024**, *11*, 2024.

[20] C. Ausilio, C. Lubrano, D. Rana, G. M. Matrone, U. Bruno, F. Santoro, *Adv. Sci.* **2024**, *11*, 1.

[21] A. Lobosco, C. Lubrano, D. Rana, V. R. Montes, S. Musall, A. Offenhäusser, F. Santoro, *Adv. Mater.* **2024**, *2409614*, 1.

[22] T. Sarkar, K. Lieberth, A. Pavlou, T. Frank, V. Mailaender, I. McCulloch, P. W. M. Blom, F. Torricelli, P. Gkoupidenis, *Nat. Electron.* **2022**, *5*, 774.

[23] L. K. Tamm, H. M. McConnell, *Biophys. J.* **1985**, *47*, 105.

[24] Y. Zhang, S. Wustoni, A. Savva, A. Giovannitti, I. McCulloch, S. Inal, *J. Mater. Chem. C* **2018**, *6*, 5218.

[25] Y. Zhang, S. Inal, C. Y. Hsia, M. Ferro, M. Ferro, S. Daniel, R. M. Owens, *Adv. Funct. Mater.* **2016**, *26*, 7304.

[26] R. M. Owens, S. Daniel, A. M. Pappa, H. Y. Liu, W. Traberg-Christensen, Q. Thiburce, A. Savva, A. Pavia, A. Salleo, *ACS Nano* **2020**, *14*, 12538.

[27] E. A. Schafer, E. Davis, Z. Manzer, S. Daniel, J. Rivnay, *ACS Appl. Mater. Interfaces* **2023**, *15*, 24638.

[28] H. Y. Liu, A. M. Pappa, T. C. Hidalgo, S. Inal, R. M. Owens, S. Daniel, *Anal. Bioanal. Chem.* **2020**, *412*, 6265.

[29] F. Corrado, U. Bruno, M. Prato, A. Carella, V. Criscuolo, A. Massaro, M. Pavone, A. B. Muñoz-García, S. Forti, C. Coletti, O. Bettucci, F. Santoro, *Nat. Commun.* **2023**, *14*, 1.

[30] C. Lubrano, U. Bruno, C. Ausilio, F. Santoro, *Adv. Mater.* **2022**, *34*, DOI 10.1002/adma.202110194.

[31] M. Magliulo, A. Mallardi, M. Y. Mulla, S. Cotrone, B. R. Pistillo, P. Favia, I. Vikholm-Lundin, G. Palazzo, L. Torsi, *Adv. Mater.* **2013**, *25*, 2090.

[32] F. Santoro, C. Lubrano, G. M. Matrone, G. Iaconis, *ACS Nano* **2020**, *14*, 12271.

[33] J. J. Maraj, E. A. Schafer, M. M. Mansour, E. A. Hussein, J. Berryman, E. Klavon, J. Rivnay, S. A. Sarles, *Adv. Electron. Mater.* **2024**, *2400526*, DOI 10.1002/aelm.202400526.

[34] Z. Lu, D. van Niekerk, A. Savva, K. Kallitsis, Q. Thiburce, A. Salleo, A. M. Pappa, R. M. Owens, *J. Mater. Chem. C* **2022**, *10*, 8050.

[35] M. Berggren, G. G. Malliaras, *Science (80-. ).* **2019**, *364*, 233.

[36] D. A. Koutsouras, L. V. Lingstedt, K. Lieberth, J. Reinholz, V. Mailänder, P. W. M. Blom, P. Gkoupidenis, *Adv. Healthc. Mater.* **2019**, *8*, DOI 10.1002/adhm.201901215.

[37] M. Naumowicz, Z. A. Figaszewski, *Biophys. J.* **2005**, *89*, 3174.

[38] G. C. Faria, D. T. Duong, A. Salleo, C. A. Polyzoidis, S. Logothetidis, J. Rivnay, R. Owens, G. G. Malliaras, *MRS Commun.* **2014**, *4*, 189.

[39] D. Regan, J. Williams, P. Borri, W. Langbein, *Langmuir* **2019**, *35*, 13805.

[40] W. Huang, D. G. Levitt, *Biophys. J.* **1977**, *17*, 111.

[41] H. G. L. Coster, *Chapter 2 - Dielectric and Electrical Properties of Lipid Bilayers in Relation to Their Structure*, Elsevier, **2003**.

[42] M. Tripathy, A. Srivastava, *Biophys. J.* **2023**, *122*, 2727.

[43] D. M. Soumpasis, *Biophys. J.* **1983**, *41*, 95.

[44] J. Yguerabide, J. A. Schmidt, E. E. Yguerabide, *Biophys. J.* **1982**, *40*, 69.

[45] M. Przybylo, J. Sýkora, J. Humpolíčová, A. Benda, A. Zan, M. Hof, *Langmuir* **2006**, *22*, 9096.

[46] P. S. Cremer, S. G. Boxer, *J. Phys. Chem. B* **1999**, *103*, 2554.

[47] C. M. Proctor, J. Rivnay, G. G. Malliaras, *J. Polym. Sci. Part B Polym. Phys.* **2016**, *54*, 1433.

[48] J. Rivnay, P. Leleux, M. Ferro, M. Sessolo, A. Williamson, D. A. Koutsouras, D. Khodagholy, M. Ramuz, X. Strakosas, R. M. Owens, C. Benar, J. M. Badier, C. Bernard, G. G. Malliaras, *Sci. Adv.* **2015**, *1*, 1.

[49] G. Valincius, T. Meškauskas, F. Ivanauskas, *Langmuir* **2012**, *28*, 977.

[50] H. M. Seeger, G. Marino, A. Alessandrini, P. Facci, *Biophys. J.* **2009**, *97*, 1067.

[51] R. A. Böckmann, B. L. De Groot, S. Kakorin, E. Neumann, H. Grubmüller, *Biophys. J.* **2008**, *95*, 1837.

[52] M. E. Villanueva, L. Bar, P. Losada-Pérez, *Colloids Surfaces A Physicochem. Eng. Asp.* **2024**, *682*, DOI 10.1016/j.colsurfa.2023.132943.

[53] H. Y. Liu, A. M. Pappa, A. Pavia, C. Pitsalidis, Q. Thiburce, A. Salleo, R. M. Owens, S. Daniel, *Langmuir* **2020**, *36*, 7325.
